# Flexible structural arrangement and DNA-binding properties of protein p6 from *Bacillus subtillis* phage φ29

**DOI:** 10.1093/nar/gkae041

**Published:** 2024-01-28

**Authors:** Martín Alcorlo, Juan Román Luque-Ortega, Federico Gago, Alvaro Ortega, Milagros Castellanos, Pablo Chacón, Miguel de Vega, Luis Blanco, José M Hermoso, Manuel Serrano, Germán Rivas, Juan A Hermoso

**Affiliations:** Department of Crystallography and Structural Biology, Institute of Physical-Chemistry “Blas Cabrera”, CSIC, 28006 Madrid, Spain; Molecular Interactions Facility, Centro de Investigaciones Biológicas “Margarita Salas”, CSIC, 28040Madrid, Spain; Departamento de Farmacología and CSIC-IQM Associate Unit, Universidad de Alcalá, Alcalá de Henares, 28871Madrid, Spain; Department of Biochemistry and Molecular Biology ‘B’ and Immunology, Faculty of Chemistry, University of Murcia, Regional Campus of International Excellence ‘Campus Mare Nostrum, Murcia, Spain; Instituto Madrileño de Estudios Avanzados en Nanociencia (IMDEA Nanociencia), Nanotechnology for Health-Care, 28049 Madrid, Spain; Department of Biological Physical-Chemistry, Institute of Physical-Chemistry “Blas Cabrera”, CSIC, 28006Madrid, Spain; Genome maintenance and instability, Centro de Biología Molecular Severo Ochoa, CSIC-UAM, 28049Cantoblanco, Madrid, Spain; Genome maintenance and instability, Centro de Biología Molecular Severo Ochoa, CSIC-UAM, 28049Cantoblanco, Madrid, Spain; Genome maintenance and instability, Centro de Biología Molecular Severo Ochoa, CSIC-UAM, 28049Cantoblanco, Madrid, Spain; Institute for Research in Biomedicine (IRB), Barcelona Institute of Science and Technology, Barcelona, Spain; Cambridge Institute of Science, Altos Labs, Cambridge, UK; Department of Structural and Chemical Biology, Centro de Investigaciones Biológicas “Margarita Salas”, CSIC, 28040Madrid, Spain; Department of Crystallography and Structural Biology, Institute of Physical-Chemistry “Blas Cabrera”, CSIC, 28006 Madrid, Spain

## Abstract

The genome-organizing protein p6 of *Bacillus subtilis* bacteriophage φ29 plays an essential role in viral development by activating the initiation of DNA replication and participating in the early-to-late transcriptional switch. These activities require the formation of a nucleoprotein complex in which the DNA adopts a right-handed superhelix wrapping around a multimeric p6 scaffold, restraining positive supercoiling and compacting the viral genome. Due to the absence of homologous structures, prior attempts to unveil p6’s structural architecture failed. Here, we employed AlphaFold2 to engineer rational p6 constructs yielding crystals for three-dimensional structure determination. Our findings reveal a novel fold adopted by p6 that sheds light on its self-association mechanism and its interaction with DNA. By means of protein–DNA docking and molecular dynamic simulations, we have generated a comprehensive structural model for the nucleoprotein complex that consistently aligns with its established biochemical and thermodynamic parameters. Besides, through analytical ultracentrifugation, we have confirmed the hydrodynamic properties of the nucleocomplex, further validating in solution our proposed model. Importantly, the disclosed structure not only provides a highly accurate explanation for previously experimental data accumulated over decades, but also enhances our holistic understanding of the structural and functional attributes of protein p6 during φ29 infection.

## Graphical Abstract

**Graphical Abstract:**
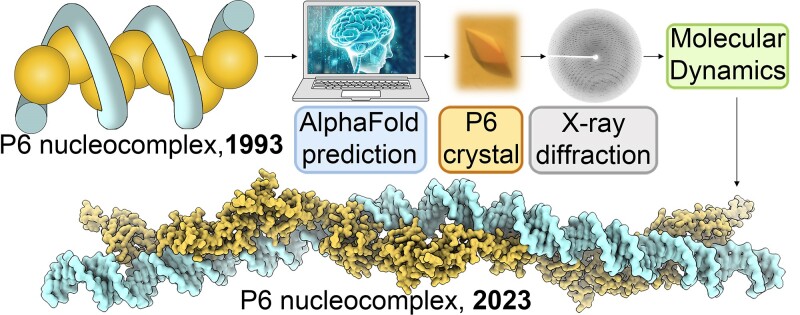

## Introduction

Bacteriophages, the most diverse and abundant biological entities on Earth (1,2), primarily belong to the order *Caudovirales* (3), which includes three families: *Myoviridae*, *Siphoviridae* and *Podoviridae* (now *Salasmaviridae* family). *Bacillus*, a genus of gram-positive, aerobic, endospore-forming microorganisms typically found in soil and decomposing plant matter, hosts a variety of phages with shared characteristics. These phages universally harbor double-stranded DNA (dsDNA) as their genetic material and have prolate icosahedral heads with tail structures. *B. subtilis* phage φ29 pertains to the *Salasmaviridae* family and to the φ29*-like* genus (currently known as *Salasvirus*), together with phages PZA, B103, Nf and GA-1 (4). While most of these phages infect *B. subtilis*, they also frequently target related species such as *B. pumilus*, *B. amyloliquefaciens*, *B. cereus*, *B. velezensis*, *B. licheniformis* or *B. thuringiensis*. When limited phage sequences were available, the φ29-like genus was classified into three groups (or sub-clusters); on the basis of serological properties, DNA physical maps, peptide maps and partial or complete DNA sequences (5–7). The first sub-cluster includes φ29 and PZA (termed B1); the second includes B103 and Nf (sub-cluster B2), and the third contains GA-1 (sub-cluster B3) (8).

Over the past decade, with the advent of next-generation sequencing techniques, several *Bacillus* phages containing proteins similar to those of φ29 and related phages, have been isolated and characterized. It is now believed that the *Salasvirus* genus should encompass at least 35 φ29-like phages, with many of them remaining unpublished and under ongoing characterization [for an in-depth genomic analysis of the newly classified *Salasmaviridae* phages and insights into their evolution and diversity, see (15)].

**Figure 1.**
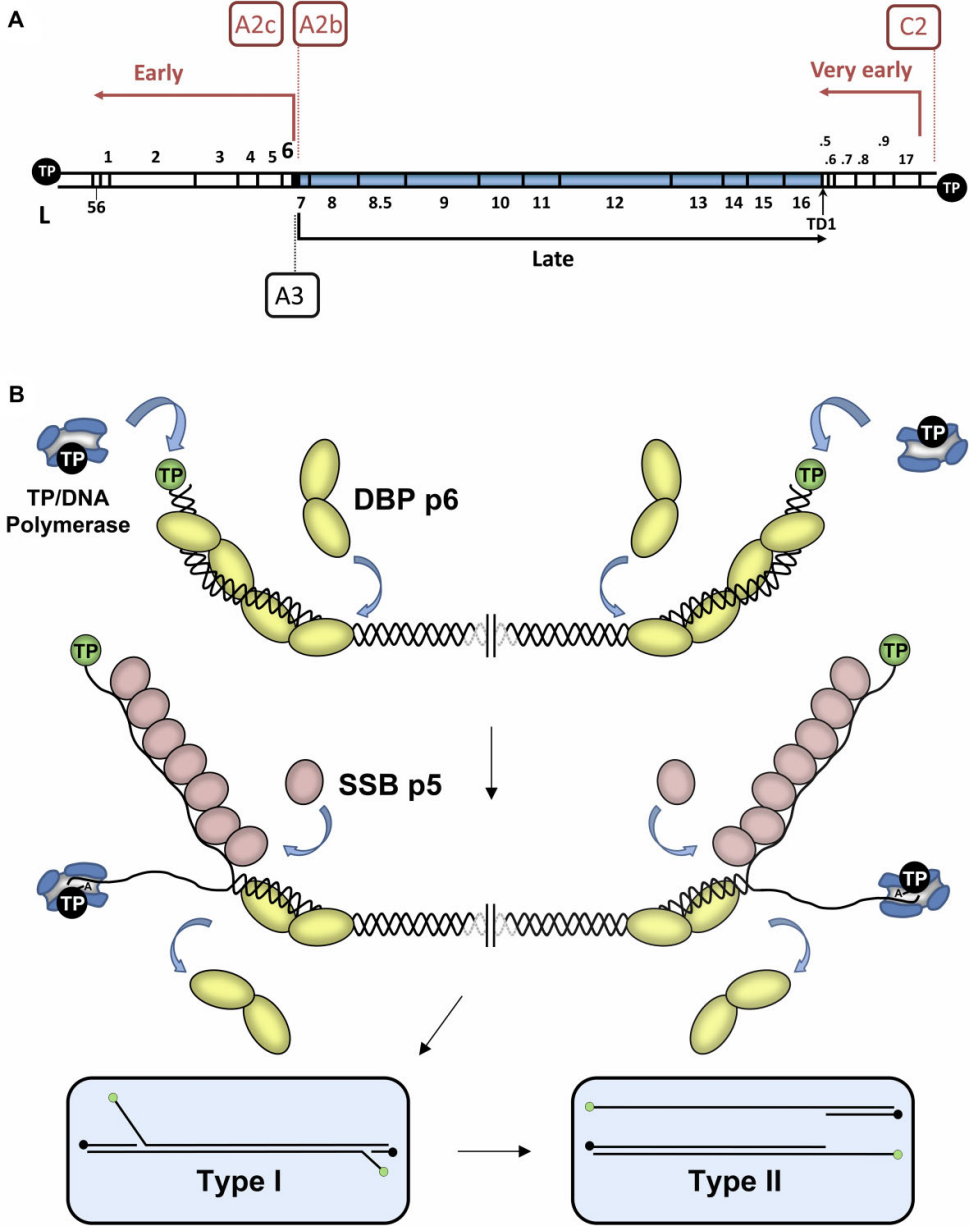
(**A**) Genetic and transcriptional map of phage φ29 genome. The positions of the various genes characterized up to date are indicated with numbers. The positions of the genes/ORFs *16.5*, *16.6*, *16.7*, *16.8* and *16.9*, located at the right side of the φ29 genome, are indicated with the numbers .5, .6, .7, .8 and .9 respectively. The main early promoters A2b, A2c and C2 are boxed in salmon colour and the late promoter A3 is boxed in black. The directions of transcription and lengths of the transcripts are indicated by arrows. TD1 stands for the position of a bidirectional transcriptional terminator. Black circles represent the terminal protein (TP) covalently linked to the 5′ DNA ends. ‘L’ and ‘R’ correspond to the left and right end of the φ29 genome, respectively. (**B**) Schematic representation of the bacteriophage φ29 DNA replication initiation mechanism. φ29 DNA replication starts non-simultaneously at both DNA ends (9). The TP/DNA polymerase heterodimer recognizes the p6-complexed replication origins and the DNA polymerase catalyzes the covalent linkage of dAMP to TP residue S232 (initiation reaction) (10,11). After a transition step (not depicted in the figure), the DNA polymerase dissociates from the TP and continues processive elongation coupled to strand displacement of the non-template strand (9). Viral protein p5 starts binding to the p6-opened DNA ends and binds cooperatively along the displaced ssDNA from each end (12). When the two replication forks meet, the type I replicative intermediate gives rise to two physically separated type II replicative intermediates. These molecules consist of full-length φ29 DNA in which a portion of the DNA starting from one end is dsDNA and the portion spanning to the other end is ssDNA (13,14). Continuous elongation by two DNA polymerases subsequently removes p5 and culminates in the complete duplication of the parental strands. Green spheres: parental TP; black spheres: primer TP; pale yellow circles: p6; blue structure: DNA polymerase; salmon ovals: p5. Linear dsDNA is shown as a double helix. DBP: dsDNA-binding protein. SSB: single-stranded DNA-binding protein.

The bacteriophage φ29 of *B. subtilis* serves as a well-established model for studying fundamental biological processes, including DNA replication, transcription and viral particle morphogenesis. φ29 harbors a linear, dsDNA of 19285 bp encompassing a minimum of 20 protein-coding genes. These genes are categorized into early and late groups based on their expression timing during the infection process. Early genes are transcribed from three main promoters: C2, A2b and A2c (Figure 1A). Late genes encode structural proteins, proteins involved in viral morphogenesis and bacterial lysis, and are transcribed from the late A3 promoter (Figure 1A). In addition, the φ29 genome features a terminal protein (TP or p3) covalently linked to the 5′ ends (the so-called parental TP, Figure 1A). The initiation of φ29 DNA replication (see Figure 1B for details) employs a protein-priming mechanism (16,17). It commences with the formation of a heterodimer between the φ29 DNA polymerase (p2) and a free TP molecule (primer TP) that recognizes the replication origins, which contains the parental TP, at both ends of the viral genome. The φ29 dsDNA-binding protein p6 (encoded by *gene 6*) forms a nucleoprotein complex at the replication origins, facilitating the initiation step of replication by potentially opening the DNA ends (18,19). Unwinding DNA at the viral genome ends promotes TP/DNA polymerase complex interaction with the template strand (20). Viral DNA replication initiation involves the covalent linkage of the first inserted nucleotide (dAMP) to the hydroxyl group of S232 on the priming TP, a reaction catalyzed by the φ29 DNA polymerase (10,11). *In vivo*, protein p6 is essential for phage DNA replication (21,22) and *in vitro*, it has been shown to stimulate both initiation and transition to elongation steps (23, 24).

φ29 protein p6 presents small size and high abundance in infected cells [about 700 000 copies/cell; 1.4 times the amount necessary to cover all the φ29 DNA molecules at mid-infection times (25)]. Because p6 interacts with the viral DNA forming a regular nucleocomplex (26,27), it has been described as a protein adapted to compact and to organize the viral genome (9). Additionally, p6 forms dimers that bind to DNA every 24 nucleotides (28) and interact with the viral DNA through the minor groove (26,29). Consequently, a protein monomer contacts the DNA every 12 bp, suggesting a model where the DNA wraps around a multimeric core of p6, forming a right-handed superhelix with approximately 63 bp per turn (20,30,27). Due to DNA wrapping, the nucleoprotein complex undergoes length reduction compared to naked DNA (27,31). Moreover, p6 possesses higher affinity for both φ29 DNA ends, which have a key role in the initiation step of replication. This binding occurs at recognition regions mapped between positions 62–125 at the right end, and between positions 46–68 at the left end (32). Notably, p6 does not recognize a specific sequence; rather, it discerns a sequence-dependent bendability pattern inherent in the recognition sites, serving as a nucleation site for protein p6–DNA complex formation (27,33).

Beyond its role in DNA replication, p6 is also implicated in transcriptional control, affecting early-late switching [for a detailed review see (34)], either independently or with assistance from the φ29 transcriptional regulator p4 (35,36). Thus, protein p6 switches off very early transcription from promoter C2 (Figure 1A), as shown by *in vivo* and *in vitro* studies, preventing RNA polymerase access to the promoter region (32,36). Moreover, the formation of the p6 nucleoprotein complex facilitates p4-mediated repression of early promoters A2b and A2c and activation of the late A3 promoter (37) (Figure 1A).

*In vivo*, p6 is able to discriminate between bacterial and viral DNA based on their different superhelicity (33). Therefore, p6 is capable of restraining positive supercoiling of the DNA *in vitro* (20,28) and binding all along φ29 DNA *in vivo* with a much higher affinity than for plasmid DNA, although binding to plasmid DNA is enhanced by decreasing the negative supercoiling (34). As a result, the lower negative superhelicity of φ29 DNA compared to the host chromosome likely renders the viral genome an appropriate target for p6 binding (19,33). Interestingly, the preferential binding of φ29 p6 to the less negatively supercoiled viral genome seems to be quite specific, since the related bacteriophage GA-1 p6, which has a highly conserved sequence (40.45% identity, Supplementary Figure S1A and Table 1), fails to demonstrate a similar binding pattern (38) and accordingly, the GA-1 p6 complex with φ29 DNA is not functional (39).

**Table 1.** Protein p6 sequences from family *Salasmaviridiae* (φ29-like) phages

Bacteriophage	Accesion number	Length (aa)	Host
φ29	YP_002004534	104	*B. subtillis*
PZA	NP_040714	96	*B. subtillis*
Nf	YP_009910722	100	*B. subtillis*
B103	NP_690639	100	*B. subtillis*
GA-1	NP_073689	93	*Bacillus* sp.
Gxv1	YP_009910665.1	104	*Bacillus* sp.
SRT01hs	YP_009910645	93	*B. safensis*
PumA1	YP_009910581	96	*B. pumilus*
PumA2	YP_009910609	96	*B. pumilus*
Karezi	YP_009910550	90	*B. thuringiensis* kurstaki
Goe1	YP_009910698	100	*B. subtillis*
Goe4	YP_009910380	99	*B. thuringiensis* kurstaki
Goe6	YP_009910336	104	*B. velezensis*
VMY22	YP_009198016	97	*B. cereus*
KonjoTrouble	YP_009910312	102	*B. thuringiensis* kurstaki
MG-B1	YP_008060113	99	*B. weihenstephanensis*
DLn1	QWT50661	96	*B. cereus*
WhyPhy	YP_010114689	96	*B. pumilus*
BSTP4	QQO90042	103	*B. subtillis*
BSTP6	QRD99837	103	*B. subtillis*
Aurora	YP_009292375	99	*B. thuringiensis* kurstaki
Juan	YP_009910272	102	*B. thuringiensis* kurstaki
Stitch	YP_009281733	99	*Bacillus* sp.
Claudi	YP_009279597	99	*B. thuringiensis* kurstaki
Thornton	YP_010114395	99	*B. thuringiensis* kurstaki
Harambe	YP_009910147	106	*B. thuringiensis* kurstaki
QCM11	YP_009910128	99	*B. cereus* group
DK1	YP_009910426	96	*B. cereus*
DK2	YP_009910473	96	*B. cereus*
DK3	YP_009910520	96	*B. cereus*
BeachBum	ARQ95226	106	*B. thuringiensis* kurstaki
RadRaab	ASU04182	99	*B. thuringiensis* kurstaki
StevenHerd11	AZF88329	99	*B. thuringiensis* kurstaki
SerPounce	YP_009910235	99	*B. thuringiensis* kurstaki
Baseball_Field	QOC56877	99	*B. thuringiensis* kurstaki

Despite decades of dedicated research on this enigmatic protein and its intricate interaction with dsDNA, no structural information is available for p6 to this day. Extensive efforts were made over years to determine its structure using X-ray crystallography and NMR techniques but to no avail. This lack of success can be attributed to the absence of structural homologs, making it challenging to design a minimal expression construct suitable for analysis. In this work, a combination of AF2 (40) predictions with crystallographic, experimental and molecular dynamic (MD) techniques, has allowed us to generate comprehensive structural models of protein p6, which provide valuable information about its oligomeric behavior and intricate interactions with dsDNA. Based on AF2 predictions, we designed rational p6 constructs, facilitating the crystallization and determination of the long-awaited protein structure in both its monomeric and oligomeric forms. Our structural models strongly correlate with previous biochemical data. Moreover, protein DNA-docking and MD simulations have allowed us to propose a highly plausible molecular model of the nucleocomplex, meeting all pre-established biochemical parameters. In parallel, the hydrodynamic properties of the nucleocomplex have been determined by analytical ultracentrifugation, providing an additional layer of validation to the model. Altogether, this work contributes significantly to filling the existing gap in structural information regarding this biological system.

## Materials and methods

### Cloning, expression, and purification of full-length protein p6 and its C-terminal deletion mutants

Full-length protein p6 (*wt*) was produced and purified as previously described (23). For p6 C-terminal deletion mutants [p6CΔ16 (residues 1–87), p6CΔ20 (residues 1–83) and p6CΔ31 (residues 1–72)], we fused the corresponding p6 amino acid sequence at its N-terminus to an 8 His-tag followed by the Small Ubiquitin-Modifier (SUMO) protein. The coding nucleotide sequence for each mutant, optimized for *Escherichia coli* expression, was synthesized by GeneScript and cloned into the pET28b plasmid at the NcoI and HindIII sites. The sequenced constructs were used to transform *E. coli* Lemo21 cells. The resulting strains were grown until an optical density at 600 nm of 0.6–0.7 was reached, and p6 mutants’ expression was induced with 1 mM IPTG (Promega) for 3 h at 28°C. All p6 C-terminal deletion mutants were purified following the same protocol. Briefly, cell pellets were resuspended in buffer p6 (20 mM Tris–HCl pH 7.5, 500 mM NaCl and 20 mM imidazole), containing lysozyme and DNase I (Roche Diagnostics Corp., Indiana, USA) to 0.1 mg/ml and 5 μg/ml, respectively. The cell suspension was sonicated on ice for 5 min at 30% amplitude. Cell lysate was centrifuged at 16 000 *g* at 4°C for 45 min. Supernatant was loaded by gravity onto a Ni^2+^-charged column (HisTrapHP, GE Healthcare) equilibrated with buffer p6. The column was washed with 20 mM imidazole in buffer p6 until protein could not be detected, and the His-SUMO C-terminal deletion mutants was eluted by gradually increasing the concentration of imidazole from 20 to 500 mM. Eluted protein was collected in 1 ml fractions and examined using SDS-PAGE. Fractions with the highest purity were pooled and dialyzed overnight against 20 mM Tris–HCl pH 7.5, 0.5 mM DTT and 300 mM NaCl at 4°C. The His-SUMO tag of the dialyzed protein was cleaved off by digestion with His-tagged Ulp1 protease at 30°C for 1 h. The undigested protein, the free His-SUMO tag, and the His-tagged Ulp1 protease were separated from digested protein by performing a second immobilized metal affinity chromatography step with a Ni^2+^-charged column. The digested p6 variants were collected in the flow-through and concentrated up to 9–10 mg/ml using a Millipore ultra-concentrator (10 kDa cutoff).

### Circular dichroism (CD) spectroscopy and thermal denaturation

CD experiments were conducted in a J-815 Circular Dichroism Spectrophotometer (Jasco Corporation) using a quartz cuvette with a 0.1-cm path length. Proteins were diluted in NaPO_4_ 20 mM pH 7.4 and NaF 50 mM buffer at different concentrations between 12 and 20 μM. For thermal denaturation experiments, the ellipticity in the range between 190 and 300 nm was followed over the temperature range of 4–90°C with heating at a rate of 2°C/min and 50 nm/min of scanning speed. The mid-denaturation temperature (*T*_m_) at 222 nm was calculated using the Origin Software (OriginLab Corporation, Northampton, MA, USA) and the Boltzmann equation for the sigmoidal fitting of the data (Equation 1):


(1) \begin{eqnarray*}y = \;\frac{{{A_{1\;}} - \;{A_2}}}{{1 + \;{e^{\left( {x - \;{x_o}} \right)/{d_x}}}}} + \;{A_2}\end{eqnarray*}

where *A*_1_ and *A*_2_ are the initial and final CD value at 222 nm in the temperature ramp, respectively, *x*_0_ is the center (*T*_m_) and *d_x_* the width. The secondary structure content was estimated using the BestSel online tool (ELTE Eötvös Loránd University, Budapest, Hungary) (41,42).

### Protein crystallization

Crystallization screenings were performed using high-throughput techniques in a NanoDrop robot and Innovadyne SD-2 microplates (Innovadyne Technologies, Inc.) with screening PACT Suite and JCSG Suite (Qiagen), JBScreen Classic 1 to 4 (Jena Bioscience), Morpheus and MIDASplus (Molecular Dimensions) and Crystal Screen I&II, SaltRX and Index HT (Hampton Research). The conditions that produced crystals were optimized with a sitting-drop vapor-diffusion method at 291 K by mixing 1μl of protein solution and 1 μl of precipitant solution, equilibrated against 150 μl of precipitant solution in the reservoir chamber. For p6CΔ31, the best crystals were obtained in a crystallization condition containing 0.1 M Bis–Tris Propane pH 7.0 and 1.3 M di-Ammonium Tartrate (Supplementary Figure S2B). For p6CΔ20, the best crystals were obtained in a crystallization condition containing 10% *w/v* PEG 8000; 20% *v/v* ethylene glycol; 0.02 M 1,6-hexanediol; 0.02 M 1-butanol; 0.02 M (*RS*)-1,2-propanediol; 0.02 M 2-propanol; 0.02 M 1,4-butanediol; 0.02 M 1,3-propanediol and 0.1 M MES/imidazole pH 6.5. In both cases, protein concentration was assayed at the concentration of 9–10 mg/ml.

### X-ray data collection, phasing and model refinement

Diffraction data were collected in the XALOC beamline at the ALBA synchrotron (Barcelona, Spain) using a Pilatus 6M detector. Protein p6CΔ31 crystals diffracted up to 1.59 Å resolution and belonged to the *P*3_1_ 2 1 space group with the unit cell parameters *a*= 59.26 Å, *b*= 59.26 Å, *c*= 41.67 Å, α = β = 90° and γ = 120°. Protein p6CΔ20 crystals diffracted up to 2.30 Å resolution and belonged to the P3_1_ 2 1 space group with the unit cell parameters *a*= 44.19 Å, *b*= 44.19 Å, *c*= 206.74 Å, α = β = 90° and γ = 120°. The collected data sets were processed with XDS (43) and Aimless (44). In the p6CΔ31 crystals, one monomer was found in the asymmetric unit, yielding a Matthews coefficient (45) of 2.21 Å^3^/Da and a solvent content of 44.78%. In the p6CΔ20crystals, two monomers were found in the asymmetric unit, yielding a Matthews coefficient of 3.05 Å^3^/Da and a solvent content of 59.66%. For p6CΔ31, the structure determination was performed with the molecular replacement method using the predicted AF2 structure for monomeric p6 in PHASER (46). For p6CΔ20, the structure determination was also performed with the molecular replacement method. In this last case, the search model used was the experimental structure of p6CΔ31. After locating two copies into the electron density, the model was manually completed using Coot (47) and subjected to several iterative refinement cycles using PHENIX (48). Statistics for the crystallographic data and structure solution are summarized in Table 2.

**Table 2.** Percent identity matrix

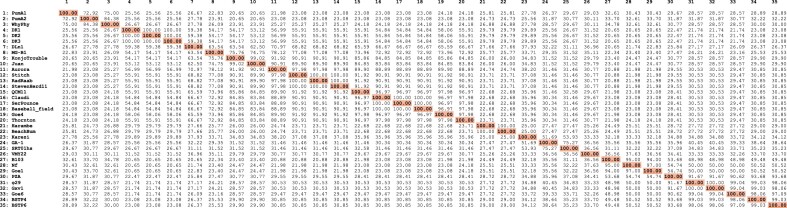

### L fragment DNA amplification

Proteinase-K (Boehringer Mannheim)-digested ϕ29 DNA was prepared as described (49). DNA–L fragment (259 bp long) was obtained by PCR amplification from genomic ϕ29 DNA using primer 1 (5′-AAA GTA AGC CCC CAC CCT CAC ATG) and primer 2 (5′-GCC CAC ATA CTT TGT TGA TTG G). The synthetic oligonucleotides were obtained from Macrogen and the Taq polymerase from Jena. The amplification conditions for the L fragment included a preheating step of 10 min at 95°C, followed by 30 cycles comprising a denaturation step of 15 s at 95°C, a hybridization step of 5 s at 53°C and an elongation step at 72°C lasting 15 s. The amplified DNA was purified using NucloSpin columns for PCR clean-up (Machery-Nagel) and eluted in 5 mM Tris–HCl pH 7.5.

### Analytical ultracentrifugation assays

#### Sedimentation velocity (SV) assays

Experiments were performed in an Optima XL-I analytical ultracentrifuge (Beckman-Coulter Inc.) equipped with both UV-VIS absorbance and Raleigh interference detection systems, using an An-50Ti rotor and 12 mm optical pass epon-charcoal standard double sector centerpieces. Samples of p6 alone (*wt* or p6CΔ20) or mixed with the L fragment [in 50 mM Tris–HCl (pH 7.5), 10 mM MgCl_2_ and 50 mM NaCl], were centrifuged at 48 000 or 42 000 rpm, respectively at 20°C. Sedimentation was followed simultaneously by Raleigh interference and absorbance at 230 nm, in the case of protein alone, or 260 nm, for the mixtures of protein and DNA–L. Differential sedimentation coefficient distributions were calculated by least-squares boundary modelling of sedimentation velocity data using the continuous distribution c(*s*) Lamm equation model as implemented by SEDFIT software (50). These experimental s values were corrected to standard conditions of water at 20°C with the program SEDNTERP (51,52) to obtain the corresponding standard s values (*s*_20,w_). The concentration dependent changes of the c(*s*) distributions corresponding to *wt* p6 and p6CΔ20 were modelled by isotherms based on the integrated weight-average sedimentation coefficients and analyzed through a monomer–dimer self-association binding model as implemented in SEDPHAT software (53).

#### Sedimentation equilibrium (SE) assays

Short columns (90 μl) SE experiments of DNA–L fragment (0.1 μM) titrated with increasing concentrations of *wt* p6 or p6CΔ20 (0.1–20 μM) were carried out at 5000 rpm and 260 nm, using the same experimental conditions and instrument as in the SV experiments. All samples were equilibrated in 50 mM Tris–HCl (pH 7.5), 50 mM NaCl and 10 mM MgCl_2_ buffer. The large size divergence between protein p6 variants (∼9.5 and 11.8 kDa for p6CΔ20 and *wt* p6, respectively) and the DNA–L fragment (∼170 kDa), together with the very low extinction of protein p6 at 260 nm, allow the analysis of the concentration gradients corresponding to DNA–L and DNA–L bound to p6, in the presence of free protein p6, which does not sediment at 5000 rpm. To obtain the corresponding baseline offsets a last high-speed run was done to deplete DNA species from the meniscus region. Weight-average buoyant molecular weights of DNA–L and DNA–L–protein p6 complexes were determined by fitting a single species model to the experimental data using the HeteroAnalysis program (54) once corrected for temperature and solvent composition with the program SEDNTERP (51). SE assays with *wt* p6 and p6ΔC20 alone were done at 15 000 rpm and analyzed as described above. The amount of protein bound to DNA-L fragment was determined from the experimental apparent buoyant mass increments, using 0.7314 cm^3^/g and 0.7414 cm^3^/g as partial specific volume for *wt* p6 and p6ΔC20, respectively, calculated from their amino acid composition by SEDNTERP. The binding isotherms built from these experimental buoyant mass increments, were modelled through an empirical three-parameter Hill plot, as implemented in SigmaPlot 11.0 software (Equation 2):


(2) \begin{eqnarray*}y = \frac{{a{x^b}}}{{{K_d}^b + {x^b}}}\end{eqnarray*}

where *y* stands for the number of proteins bound per DNA–L, *a* denotes the maximum number of proteins bound at saturation, *x* is the total concentration of protein, *K_d_* is the concentration of half-maximal binding, and *b* is an empirical cooperativity parameter.

### Hydrodynamic modeling

Theoretical sedimentation coefficients for each atomic entity were computed using the bead modelling software HYDROPRO (55). A primary bead radius of 2.9 Å was assigned to represent each atom. The sedimentation coefficients were calculated under standard conditions (293 K and water solvent). The Molecular weights and partial specific volumes (vbar) for each model were determined based on the protein and DNA sequences using Sednterp (52). For the variant p6CΔ20, the primary models for the atomic detailed structures were constructed in pdb format using available atomic coordinates from crystallographic data or predicted models from AF2. For the p6CΔ20–DNA complexes, two approaches were employed to model the nucleocomplex. Firstly, for the p6CΔ20 structure derived from X-ray crystallography, manual constructs were generated using the software Chimera v1.16 (56). These models were assembled by combining experimental p6 structures from the p6CΔ20 oligomer with 12 bp DNA fragments corresponding to each p6 monomer. Secondly, for the AF2-derived p6CΔ20 oligomer structure in complex with DNA, the coordinates of the resulting complex after protein p6 docking into the DNA followed by MD simulations (see below), were directly utilized. In this last case, a repetitive sequence of 24 bp was employed to generate the nucleocomplex. In both cases, the resulting models were then treated as repetitive units, resulting in the construction of final structures consisting of 22 p6 protein monomers and a 259 bp DNA.

### Model building of DNA linear duplexes and their complexes with p6 proteins

he all-atom DNA models were built with the aid of the MCDNA web server (https://mmb.irbbarcelona.org/MCDNA/), a component of the Multiscale Genomics project (https://www.multiscalegenomics.eu/MuGVRE/). The initial models of the protein-DNA complexes were generated with the aid of the pyDockDNA web server (57). A high degree of convergence of the best-scoring models toward very similar structures was obtained when the presence of K2 and R6 at the binding interface was defined as the only external restraint (58) to help score the docking solutions together with the calculated electrostatic and desolvation binding energies. The molecular graphics program PyMOL v.1.8 (Schrödinger, LLC. 2015) and ChimeraX v.1.5 (59) was employed for molecular editing, visualization and figure preparation.

### Model building of the DNase I–DNA complexes

The X-ray crystal structures of bovine DNase I in complex with the self-complementary nicked octamer d(GCGATCGC)_2_ (PDB 2DNJ) (60) and the uncleaved d(GGTATACC)_2_ duplex (PDB 1DNK) (61), solved at 2.0 and 2.3 Å resolution, respectively, were used for modeling DNase I bound to the minor grooves of two different ds DNA molecules containing either 5′- CGCGATCGCGATGCGC-3′ or 5′-CCTAATATCGACATAATCCGTCGAC-3′ in the Watson strand (with the cleavage site underlined) and the complementary sequence in the Crick strand. For correct placement of the catalytic Mg^2+^ and its coordination sphere in the active site, the 1.95 Å resolution crystal structure of human DNase I in complex with magnesium and phosphate ions (PDB 4AWN) (62) served as a template for best-fit superposition. The two conserved disulfide bridges between C101-C104 and C173–C209 were defined, and a Ca^2+^ ion was located at the calcium-binding loop made up by D201, T203, T205 and T207 (60).

### Molecular dynamics (MD) simulations

The unrestrained MD simulations were run in explicit physiological saline solution under periodic boundary conditions using the ff14SB AMBER force field and PARMBSC1 modifications (63) for DNA, essentially as described (64). Briefly, electrostatic interactions were represented using the smooth particle mesh Ewald method with a grid spacing of 1 Å and the cutoff distance for the non-bonded interactions was 9 Å. The SHAKE algorithm was applied to all bonds involving hydrogens so that an integration step of 2.0 fs could be used. The simulation protocol made use of the *pmemd.cuda_SPFP* engine implemented in AMBER 18 (65) running on single Nvidia GTX 1080 RTX2080Ti GPUs. First, solvent molecules and counterions were allowed to redistribute around the positionally restrained solute (5 kcal mol^−1^ Å^−2^) using energy minimization and the resulting systems were progressively heated from 100 to 300 K during 0.1 ns using the same restraints. Then, the systems were equilibrated at 300 K for 2.5 ns in the presence of weak restraints on the proteins’ Cα atoms and further simulated in the absence of any restraints for at least 250 ns during which system coordinates (‘wrapped’ into the primary box) were collected every 5 ns for further analysis.

### Analysis of the MD trajectories

Distances and angles were monitored by making use of the *cpptraj* module in AmberTools (66) whereas estimations of the solvent-corrected protein-protein and protein-DNA binding energies, as well as their per-residue decompositions into van der Waals, coulombic, apolar and desolvation contributions were provided by the MM-ISMSA software (67). The DNA conformational features were monitored using the software CURVES+ (68), as implemented in the NAflex web server (69).

## Results and discussion

### Sequence comparison of the protein p6 family

According to the Pfam database (70), protein p6 is classified into the p6 family (PF17548). This family consists of ∼100-residue proteins with a single predicted ‘*p6 domain*’, found in tailed bacteriophages of the order *Caudovirales*. For exploration of p6 family characteristics, we performed a multiple sequence alignment (Supplementary Figure S1A) using all available p6 protein sequences (Table 1, updated on February 2021) from the genus *Salasvirus* (φ29-like phages). These 35 proteins sequences, exhibiting identities ranging from 20 to 100% (see Table 2), share identical predicted secondary structure elements, indicating a conserved fold. The sequence alignment revealed a highly conserved N-terminal region spanning residues 1–84 [according to the traditional numbering of φ29 protein p6, where the second Ala residue is designated position number one due to post-synthesis removal of the initial Met residue *in vivo* (23)]. This homologous region corresponds to the unique putative folded domain present in this protein family (see next section) and clusters all the few residues that are absolutely conserved among all sequences analyzed, encompassing K2, R6, G37, A44, V59 and F76. The importance of these positions for the protein's structure and function will be discussed in subsequent sections. In contrast, the C-terminal part (residues 85–103) exhibits considerable sequence variability, diminishing the alignment's quality. This region consistently contains several acidic residues and varies in length, spanning from 10 residues (phage SRT01hs) to 21 (phages Harambe and BeachBum), depending on the phage. Other very highly conserved residues include T11, Y69 and A80. According to predictions from two meta disorder prediction programs [PONDR-FIT (71) and DISOPRED2 (72)], this particular segment is expected to be disordered.

### The three-dimensional structure of φ29 protein p6 monomer reveals a novel fold

**Figure 2.**
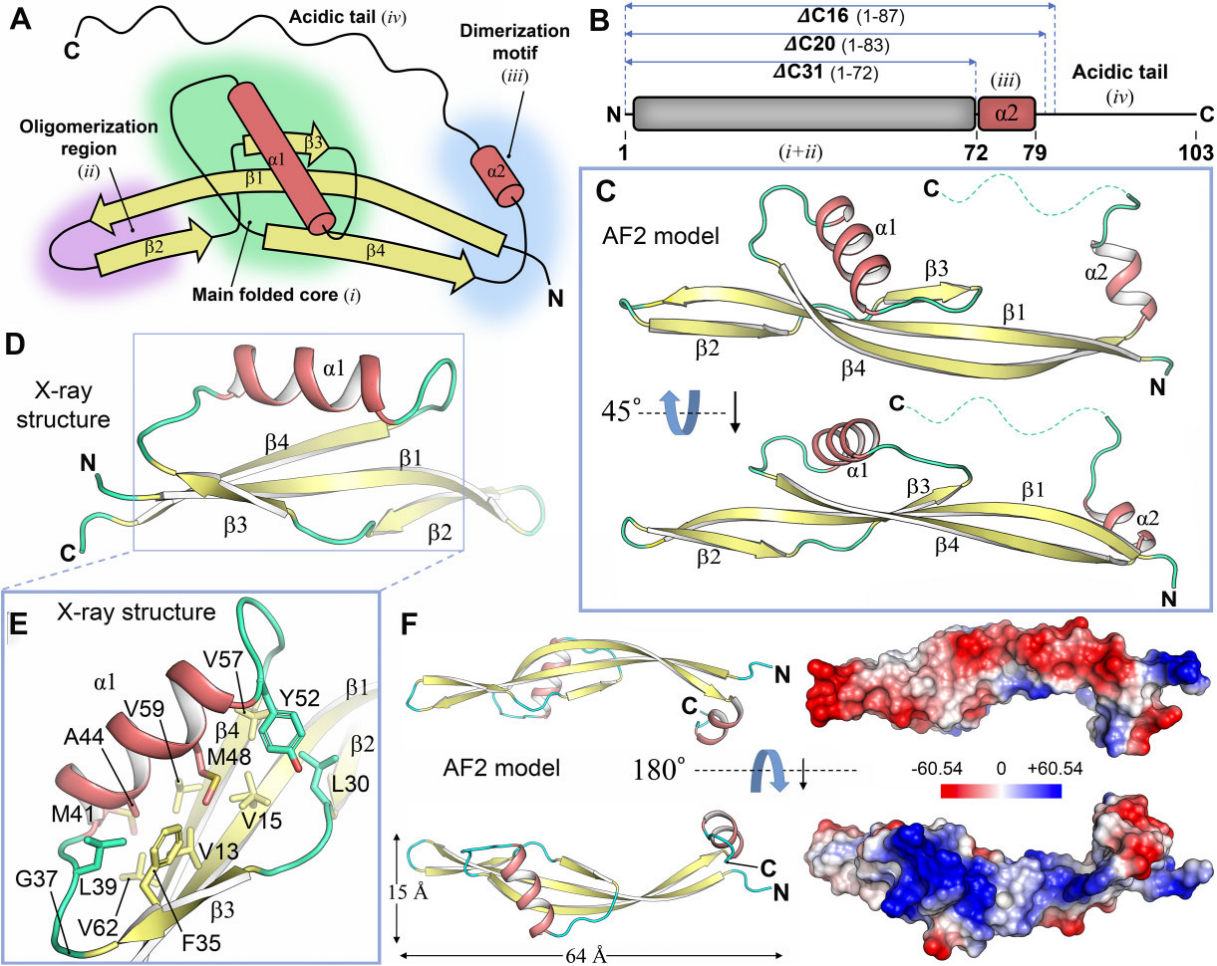
Structure prediction and crystallographic structure of monomeric φ29 protein p6. (**A**) Topological diagram of protein p6 monomer based on AF2 predictions. Labeling and color-coded representation highlight the distinct secondary structure elements, with pale yellow indicating β-strands (depicted as arrows) and salmon representing α-helices (displayed as cylinders). (**B**) Schematic representation introducing the p6 constructs designed, featuring the three C-terminal truncated proteins that were successfully expressed and purified. The p6CΔ16 and p6CΔ20 variants entail the removal of the last 16 and 20 residues, respectively. The p6CΔ31 variant encompasses the removal of the last 31 residues, including the α2, a key element involved in dimerization (see next section). (**C**) AF2 model for the monomeric protein p6. The cartoon structure is displayed in two orientations at 45° of each other, following the same color code displayed in panel A. In addition, coil regions are depicted in cyan. The acidic C-terminal tail is represented as a dashed line. (**D**) Crystal structure of p6CΔ31 at ∼1.6 Å resolution following the same representation and color code depicted in C. (**E**) Residues that build up the hydrophobic compact core of protein p6 are displayed based on their arrangement in the crystallographic structure of p6CΔ31. This region corresponds to the boxed area shown in panel D. (**F**) Electrostatic-potential surface of the predicted p6 monomer shown in two orientations differing by 180°. The color key (blue, positive and red, negative) denotes the Poisson–Boltzmann electrostatic-potential surface (color bar range ± 60.54 kT/e). The right panel aligns with the ribbon representation shown on the left. N, amino-terminus; C, carboxy-terminus.

The AF2 (40) model of full-length φ29 protein p6 is confidently assigned as an α + β structure (17.5% α, 46.3% β; Supplementary Figure S2D). Overall, the model shows an elongated shape (15 × 15 × 64 Å) (Figure 2F) with the polypeptide chain organized into four different regions (Figure 2A): (i) a well-defined main folded core (residues 1–16 and 29–72); (ii) a β-hairpin involved in oligomerization (residues 17–28, see later); (iii) an α-helical dimerization motif comprising a short α-helix (α2, residues 73–79, see later) and (iv) a disordered C-terminal tail of 20 residues, which corresponds to 19.4% of the total residues of the protein. It's worth noting that the C-terminal tail is assigned low confidence in the AF2 model (Supplementary Figure S2D). An interesting feature is the heterogeneous distribution of charges across the protein p6 model's surface. Specifically, positive charges cluster on one side of the monomer, while negative charges are primarily situated on the opposite face of the monomer, extending into the C-terminal tail region (Figure 2F). To evaluate the reliability of AF2’s structural prediction, we utilized Circular Dichroism (CD) spectroscopy to experimentally ascertain the secondary structural elements in protein p6 (Supplementary Figure S3). The analysis confirmed that p6 exhibits an α + β protein architecture, with a significant β component. Notably, we observed a reasonable correlation between the experimental CD spectra and the predicted secondary structure content. Specifically, the experimental data showed 12.2% α-helices and 35.5% β-sheets, whereas the predictions indicated 17.5% α-helices and 46.3% β-sheets. These findings prompted us to systematically design several p6 constructs with targeted C-terminal deletions (Figure 2B). In a first attempt, we removed the final 31 residues (encompassing the α2 together with the C-terminal disordered acidic tail) with the aim of generating a minimal truncated protein fragment suitable for crystallization. After purifying the resulting variant, denoted as p6CΔ31 (Supplementary Figure S2A), we efficiently obtained well-diffracting crystals (Table 3, Supplementary Figure S2B and C) that permitted its structural determination at ∼1.6 Å resolution (experimental electron density is shown in Supplementary Figure S2E). A comparison between the experimental p6CΔ31 structure and the AF2 prediction revealed nearly identical backbone structures, with a *rmsd* of only 0.664 Å across 53 pruned atom pairs (Supplementary Figure S2D). The folded core of p6CΔ31 assembles into a concave four-stranded antiparallel β-sheet (β1–β4). The central feature of this fold comprises the β1 strand interacting in an antiparallel orientation with the adjacent β2 and β4 at one side, and with β3 on the other. Furthermore, a 12 residues-long α-helix (α1) bridges β3 and β4 strands and packs against β1, β3 and β4. This spatial arrangement builds up a compact and well-ordered core (Figure 2D and E) facilitated by the burial of numerous hydrophobic residues (V13, V15, L30, F35, L39, M41, A44, M48, Y52, V57, V59 and V62). Hydrogen-bonds further reinforce the structural integrity of this core. We performed a conservation analysis of amino acid positions with the program Consurf (73) (Supplementary Figure S1B), which revealed that residues from the hydrophobic core V59 and A44 are absolutely conserved within the p6 family, underscoring their pivotal role in maintaining structural integrity and proper folding. G37, another strictly conserved residue, is located immediately after the β3 strand and facilitates a significant kink in the protein backbone prior to the assembly of α1 (Figure 2E). In the crystallographic structure of p6CΔ31, residues 1–8 and 68–72, which would extend β1 and β4 strands, respectively, are not visible despite their inclusion in the construct's sequence. This likely results from the absence of stabilizing interactions provided by the α2 within this region when the protein is in a dimeric state (see next section). Nevertheless, according to the AF2 prediction for the full-length monomeric p6 (Figure 2A and C), the polypeptide chain continues from the last β strand (β4) of the folded core, promptly transitioning into the short α2 helix (residues 75–79). The α2 is almost immediately followed by the C-terminal disordered acidic tail spanning residues 80–103. In the case of φ29 protein p6, the C-terminal region comprises ten negatively-charged residues.

**Table 3.** Crystallographic data collection and refinement statistics*

	p6ΔC31	p6ΔC20
**Data collection**		
Wavelength (Å)	0.97926	0.97925
Space group	*P* 3_1_ 2 1	*P* 3_1_ 2 1
Unit cell *a, b, c* (Å)	59.26, 59.26, 41.67	44.19, 44.19, 206.74
Unit cell α, β, γ (º)	90, 90, 120	90, 90, 120
*T* (K)	100	100
X-ray source	Synchrotron	Synchrotron
Resolution range (Å)	41.67–1.59	41.35–2.30)
	(1.62–1.59)	(2.38–2.30)
Unique reflections	11 565 (550)	11 226 (1059)
Completeness (%)	99.30 (98.10)	100.00 (100.00)
Multiplicity	19.5 (20.4)	18.8 (19.2)
*R*_merge_ ^a^	0.040 (1.026)	0.086 (1.035)
*R*_pim_ ^b^	0.010 (0.231)	0.020 (0.239)
*<I/σ(I)>*	37.3 (3.6)	24.0 (3.5)
CC1/2	1.00 (0.94)	0.99 (0.89)
**Refinement**		
Resolution range (Å)	32.35–1.59	14.69–2.3
*R*_work_ */R*_free_ ^c^	0.2144/ 0.2237	0.2438/ 0.2761
No. atoms		
Protein	493	1340
Water	25	13
Ligand	-	-
**R.m.s. deviations**		
Bond length (Å)	0.009	0.003
Bond angles (°)	1.06	0.84
**Ramachandran** Favored/outliers (%)	98/0	98/0
Monomers per AU	1	2
**Average *B*-factor** Macromolecules	38.35 38.09	73.89 74.01
Ligands	-	-
Solvent	43.36	60.95
**PDB code**	8PW2	8PW4

*Values between parentheses correspond to the highest resolution shells.

a *R*_merge_ = Σ_*hkl*_ Σ_*i*_ | *I_i_*(*hkl*) – [*I*(*hkl*)] |/Σ_*hkl*_Σ_*i*_I_*i*_(*hkl*), where Σ*_i_I_i_*(*hkl*) is the *i*th measurement of reflection *hkl*, [*I*(*hkl*)] is the weighted mean of all measurements.

b *R*_pim_ = Σ_*hkl*_[1/(*N* – 1)]^1/2^ Σ_*i*_ | *I_i_*(*hkl*) – [*I*(*hkl*)] |/Σ_*hkl*_Σ_*i*_I_*i*_(*hkl*), where Σ*_i_I_i_*(*hkl*) is the *i*th measurement of reflection *hkl*, [*I*(*hkl*)] is the weighted mean of all measurements and *N* is the redundancy for the *hkl* reflection.

c *R*_work_/*R*_free_ = Σ_*hkl*_| *F*_o_ – *F*_c_ |/Σ_*hkl*_ | *F*_o_ |, where *F*_c_ is the calculated and *F*_o_ is the observed structure factor amplitude of reflection *hkl* for the working/free (5%) set, respectively.

A search for proteins structurally related to φ29 protein p6 with servers DALI (74), PDBeFold (75), CATH (76), COFACTOR (77), SMART (78) and ProFunc (79), yielded no statistically significant matches. For this reason, we propose that the overall p6 structure is unique, presenting a novel fold that does not resemble any structure currently deposited in the PDB.

### Architecture of the p6 dimer

Oligomerization of p6 is well-known to play a crucial role in its physiological function (34). Consequently, our next objective was to explore the structural details of p6 dimerization and oligomerization, aiming to understand the mechanisms governing these processes. To achieve this, we successfully produced and purified a second p6 variant lacking the last 16 C-terminal residues (denoted as p6CΔ16, Figure 2B). This truncated variant contains the core region (i) and the oligomerization region (ii) (both also found in p6CΔ31), but in addition encompasses the dimerization region (iii) and the first four residues of the acidic tail (region iv) (Figure 2A).

**Figure 3.**
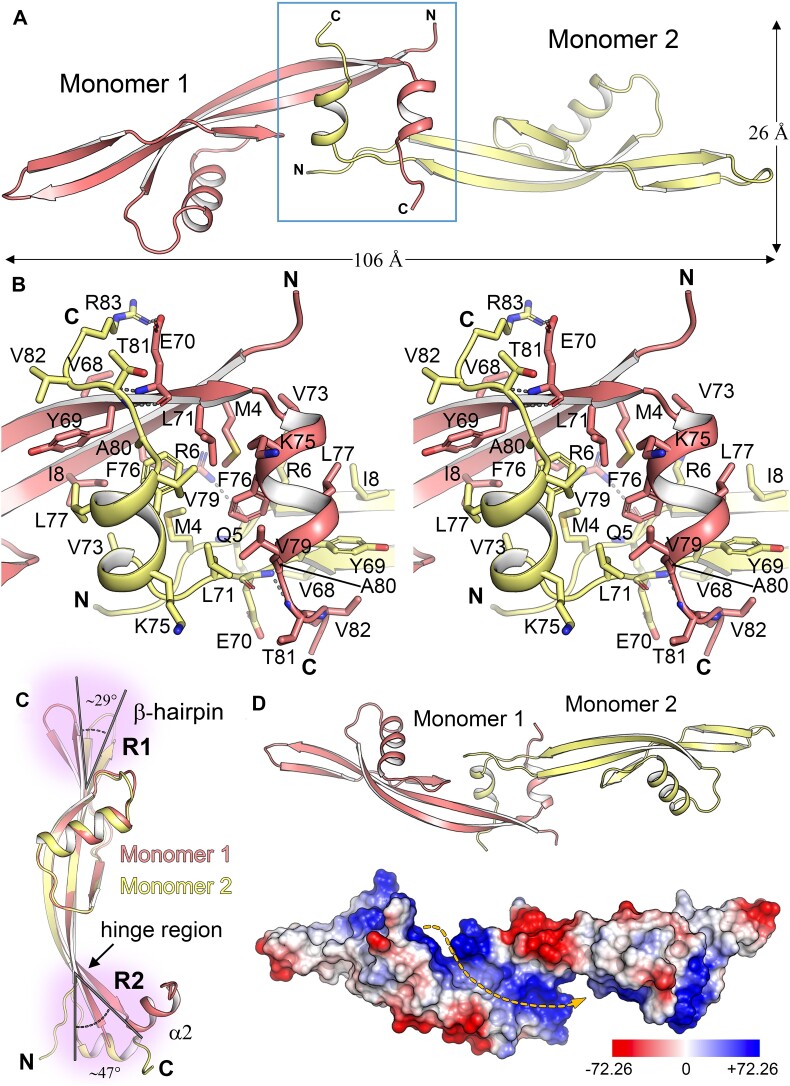
Crystal structure of the p6CΔ20 dimer. (**A**) View of φ29 protein p6CΔ20 dimer along its longitudinal axis represented in cartoon. Monomer 1 of the dimer is colored salmon, and Monomer 2 is colored pale yellow. (**B**) Stereo view of the DI. Detailed view of the interaction between Monomer 1 α2 and Monomer 2 α2 is shown, and corresponds to the boxed area shown in panel A. The relevant residues involved in dimerization are shown as capped sticks, while gray dotted lines indicate polar contacts. (**C**) Structural superposition of both monomers within the p6CΔ20 dimer is presented, with two regions exhibiting notable structural variability highlighted in purple and labeled as R1 and R2. See main text for details. (**D**) Upper panel shows the same cartoon representation of the p6CΔ20 dimer presented in A but rotated 180° along the longitudinal axis. Lower panel shows the electrostatic-potential surface of the p6CΔ20 dimer in the same orientation as the D upper panel. The color key (blue, positive and red, negative) shows the Poisson-Boltzmann electrostatic-potential surface (color bar range ± 72.26 kT/e). The yellow dashed line highlights the basic path surrounding the dimer along its longitudinal axis. N, amino-terminus; C, carboxy-terminus.

Such a design was motivated by previous findings indicating enhanced DNA binding capabilities of this specific variant compared to the *wt* (80). While the p6CΔ16 variant produced crystals in different conditions, they consistently exhibited limited diffraction quality (∼6–7 Å) and pronounced anisotropy, hampering structural determination. In a third attempt, we further truncated the initial four residues of the C-terminal tail, resulting in the p6CΔ20 variant (Figure 2B), which readily yielded well-formed crystals, facilitating the acquisition of high quality-diffraction data (Table 3). The 3D structure of p6CΔ20 was solved at 2.3 Å resolution and revealed the presence of two p6 monomers within the asymmetric unit (Figure 3A). Both monomers assemble in an elongated *tail-to-tail* manner, forming a p6 dimer with maximum dimensions of 26 × 26 × 106 Å. Each monomer intricately interlocks with its partner through the α2 helix from the dimerization motif (Figure 3A). Consequently, the main dimerization interface (DI) results from the precise packing of the α2 helices from both protomers. These helices adopt an antiparallel orientation, with a crossing angle of ∼47°. A similar dimerization motif involving two short helices has previously been observed in the *histone-like* nucleoid structuring (H-NS) protein (81).

According to PDBePISA (82) analysis, the interface between the p6 monomers exhibits a significant complex formation significance score (CSS) of 1.0, indicating the essential role played by the interface in mediating dimer formation. Dimerization occurs via an extensive hydrophobic interface (Figure 3B), covering ∼1000 Å^2^ of accessible surface area (ASA) including 15% of the total ASA of each subunit. The hydrophobic contact network extends along the two α2 helices involving residues L71, V73, K75 (aliphatic part), F76, L77 and V79 from each monomer. The tight packing between helices is further reinforced by additional hydrophobic contacts with residues located at the beginning of β1(M4, R6 [aliphatic part] and I8) and at the end of β4 (Y69 and L71) from both monomers. The role of F76 in dimer stability appears to be crucial, as it is fully buried within the hydrophobic interface. The relevance of F76 is underscored by its absolute conservation among all φ29-like phages (Supplementary Figure S1A and C). Y69 (corresponding to F in Karezi and PumA1 phages) and A80 (corresponding to G in PumA2 and WhyPhy phages) are additional highly conserved residues deeply embedded within the hydrophobic DI (Supplementary Figure S1A and C). Finally, a limited number of intermolecular polar contacts involving E70/R83, E70/T81, R6/N5 and K75/V79 also contribute to overall stabilization of this interaction network.

In the absence of structural information, the role of specific residues in p6 self-association was previously studied by generating random variants using degenerate PCR (80). Among these variants, only I8T replacement dramatically reduced its self-association capacity by at least 10-fold (80). With the p6CΔ20 structure reported herein, it is now evident that I8 is situated at the DI and contributes to α2 helix stabilization in the adjacent p6 monomer through hydrophobic interactions (Figure 3B).

Another variant with valine replacing alanine at position 44 showed altered but not completely impaired dimerization capacity (80). Since this position is within the folded core and distant from the dimerization interface (DI), we conducted MD simulations (Supplementary Figure S4) to investigate its role in the dimerization process, including *wt* p6 and p6I8T as controls. The simulations revealed a decrease in the dimerization energy of both variants, with a more pronounced effect in p6I8T, as expected. The substitution of Alanine with Valine in the p6A44V variant introduces additional volume, requiring the residue to accommodate itself within the folded core and consequently displacing surrounding residues. This perturbation results in a less stable structure of the p6 monomer that impacts dimerization (Supplementary Figure S4). Furthermore, N-terminal deletion mutants of protein p6, specifically p6NΔ5 and p6NΔ16, displayed impairments in self-association (see Supplementary Figure S5); with p6NΔ5 affecting dimer formation and p6NΔ13 completely abolishing it (80). Analyzing the p6CΔ20 structure, it can be inferred that both N-terminal deletions remove a portion of the β1 that contributes to dimerization by buttressing the interaction between the two α2 helices from both monomers.

Superimposition of the two chains of the p6CΔ20 dimer (by aligning the main folded core) revealed notable differences primarily concentrated in two regions. The first one (R1, corresponding to region *ii* on Figure 2A) shows a significant displacement of the β-hairpin in Monomer 1 compared to Monomer 2 (with a tilt of ∼29°, Figure 3C), indicating substantial structural plasticity in this region, crucial for p6 oligomerization as discussed in the next section. The second region (R2) involves the beginning of β1 and the end of β4, located just before α2. In Monomer 2, this region is kinked and displaced ∼47° compared to Monomer 1 and constitutes another source of significant structural plasticity that defines a ‘*hinge region*’ [predicted by the server HINGEprot (83)], involving residues E7 and E67 from β1 and β4; respectively. Remarkably, the β-sheet geometry in this region of Monomer 2 is disrupted, resulting in a coil conformation (Figure 3A and C), leading to a 7% reduction in the total β-sheet content. These motions around the hinge region resemble rotations around an articulated joint and profoundly impact the three-dimensional arrangement of the p6 oligomer (see below). Additionally, the iMODS server (84) was employed to explore the collective motions of the two monomers of the p6CΔ20 dimer and generate a feasible transition pathway between the two structures, illustrating the most likely motions occurring within the p6CΔ20 monomer structure (see Movie S1).

We also investigated the dimeric arrangement of p6CΔ20 using AlphaFold-Multimer (AF-Multimer) (85), a specialized AlphaFold tool for predicting the oligomeric states and multi-chain protein complexes with known stoichiometry. In agreement with the crystallographic structure, the predictions consistently revealed that the p6CΔ20 dimer assembles in a *tail-to-tail* fashion (Supplementary Figure S6). However, the main difference observed in the predicted dimeric structure from AF-Multimer is the presence of a strict 2-fold symmetry axis between the two monomers, which is only approximate in the crystal structure. In the latter, the beginning of β1 and the end of β4 in Monomer 2 exhibit a kinked conformation compared to Monomer 1, thereby disrupting the two-fold symmetry. Thus, in the AF-Multimer structure, strict binary symmetry is present, whereas in the crystal structure, it is only approximate 2-fold symmetry due to the flexibility of the previously described R1 and R2 regions. The structural implications of these differences will be discussed later. Protein p6 self-association studies carried out by sedimentation equilibrium (SE) in the absence of DNA, revealed that the global hydrodynamic behavior of the protein p6 monomer and dimer deviates from that of a rigid globular protein. This deviation indicates a prolate ellipsoidal shape (25), consistent with the observed structures reported here. These studies also revealed that the thermodynamic parameters associated with the dimerization of protein p6 involved a reduction in the water-accessible non-polar surface area, in agreement with the hydrophobic nature of the DI described earlier.

A notable characteristic of the p6CΔ20 dimer is the extension of the positive molecular electrostatic potential (MEP) from one monomer with the positive MEP of the other monomer (Figure 3D). As a consequence of this arrangement, the positive MEP surface defines a helical basic path along the longitudinal axis of the dimer and very likely serves as the binding site for dsDNA, as will be discussed later.

### Oligomeric structure of protein p6

**Figure 4.**
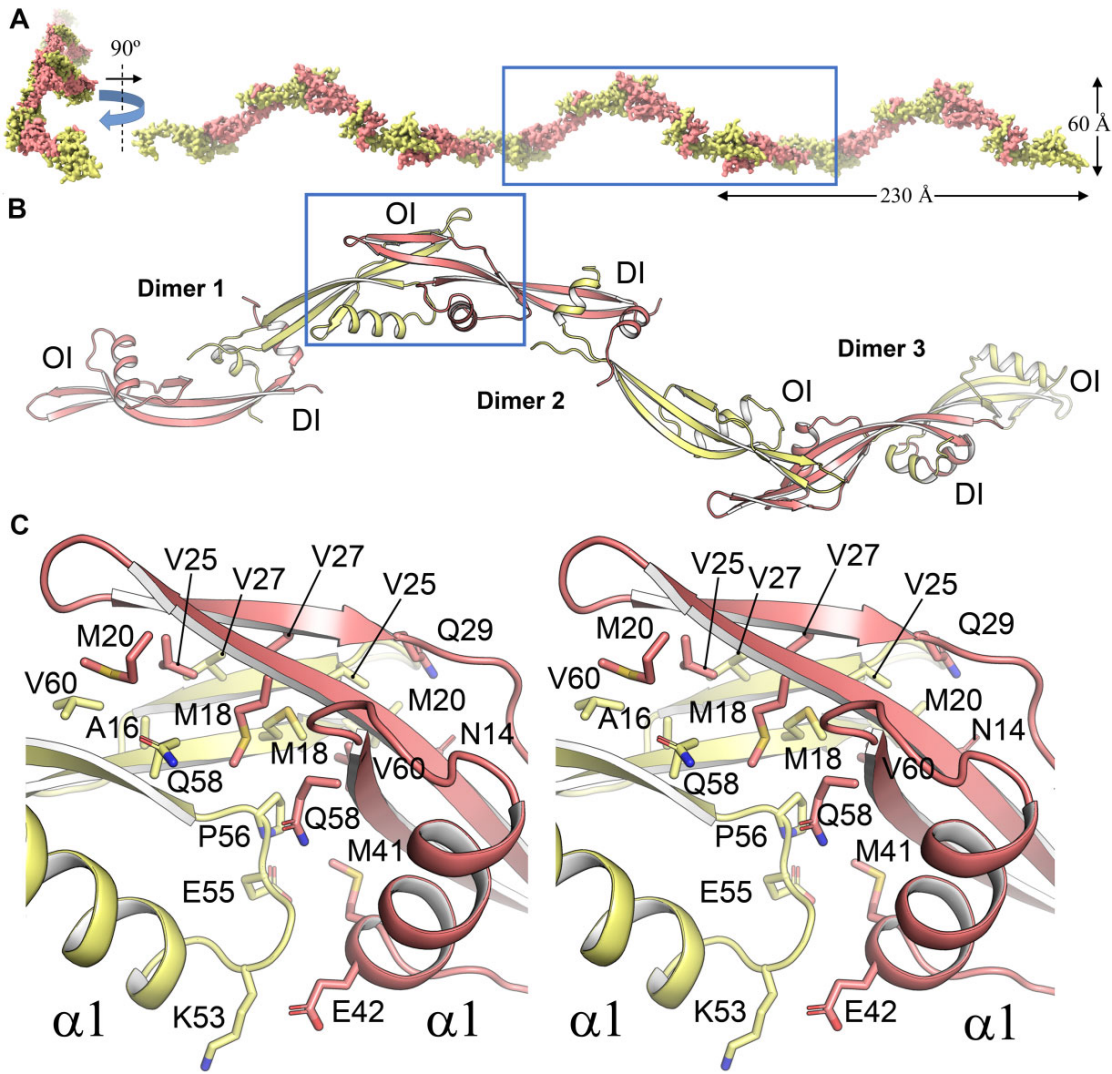
Superhelical architecture and structural basis for p6 oligomerization in the crystal lattice. (**A**) 90º views of the p6CΔ20 superhelix formed by a chain of *head-to-head* and *tail-to-tail* molecules. The orientation of the molecules, depicted as surfaces, is derived from the crystal lattice. Each monomer from a dimer is color-coded as salmon (Monomer 1) and pale yellow (Monomer 2). (**B**) Elaborate arrangement of the p6CΔ20 filament, illustrating the oligomeric configuration with monomers displayed in a cartoon representation. This depiction corresponds to the enclosed region highlighted in A. DI, dimerization interface; OI, oligomerization interface. (**C**) Stereo view of the OI, providing a detailed perspective of the interaction between Monomer 1 (salmon) and Monomer 2 (pale yellow), focusing on the enclosed region featured in panel B. Relevant residues involved in oligomerization are depicted as capped sticks.

Because protein p6 carries out its functions as homo-oligomers, knowledge of the protein's oligomeric structure is essential for atomic-level understanding of its biological functions. Interestingly, crystal packing analysis of p6CΔ20 structure unveiled that the protein crystallized as an oligomer. In this oligomer, the p6CΔ20 dimers are the building blocks that create a chain of linked dimers in a *head-to-head* fashion. This arrangement results in a superhelix with six p6CΔ20 molecules per turn, measuring ∼60 Å in diameter and 230 Å in height. The superhelix extends throughout the crystal, following the P 3_1_ screw axis (Figure 4A). This superhelical architecture facilitates the association of dimers into oligomers through two distinct structural regions, separated by ∼50 Å. One region is involved in oligomerization, while the other region mediates dimerization. Consistent with this arrangement, SE analysis demonstrated that in the absence of DNA, p6 exists in a monomer-dimer-oligomer equilibrium, with the dimerization constant differing significantly from the oligomerization constant (25). Furthermore, studies on p6 binding to concatemeric DNA sequences revealed a preference for an asymmetric dimer binding site, supporting the *head-to-head* orientation between the monomers within each dimer (86).

According to PDBePISA, the oligomerization interface (OI) is identified as a stable and functionally significant interaction region, with a CSS of 1.0. The assembly of the protein oligomer involves the packing of the β-hairpin region of each monomer against the β-hairpin region of its neighboring monomer, resulting in an exclusively hydrophobic interaction network without polar contacts (Figure 4B and C). Consequently, the α1 helices from each protein monomer are in close proximity due to protein oligomerization. Figure 4C shows a detailed view of the residues involved in oligomerization. The primary contact area encompasses ∼1000 Å^2^ of ASA, which accounts for approximately 14% of the total ASA of each subunit. To understand the role of specific amino acid groups in protein association, previous investigations examined the influence of ionic strength on the self-association of (25). These studies revealed that charged residues do not appear to significantly affect the formation of higher-order p6 association states, aligning with the exclusively hydrophobic nature of the aforementioned OI. Taken together, the structural characteristics of the p6 filament and the proposed dimerization/oligomerization mode described above offer a compelling rationale for understanding the mechanism of protein self-association.

**Figure 5.**
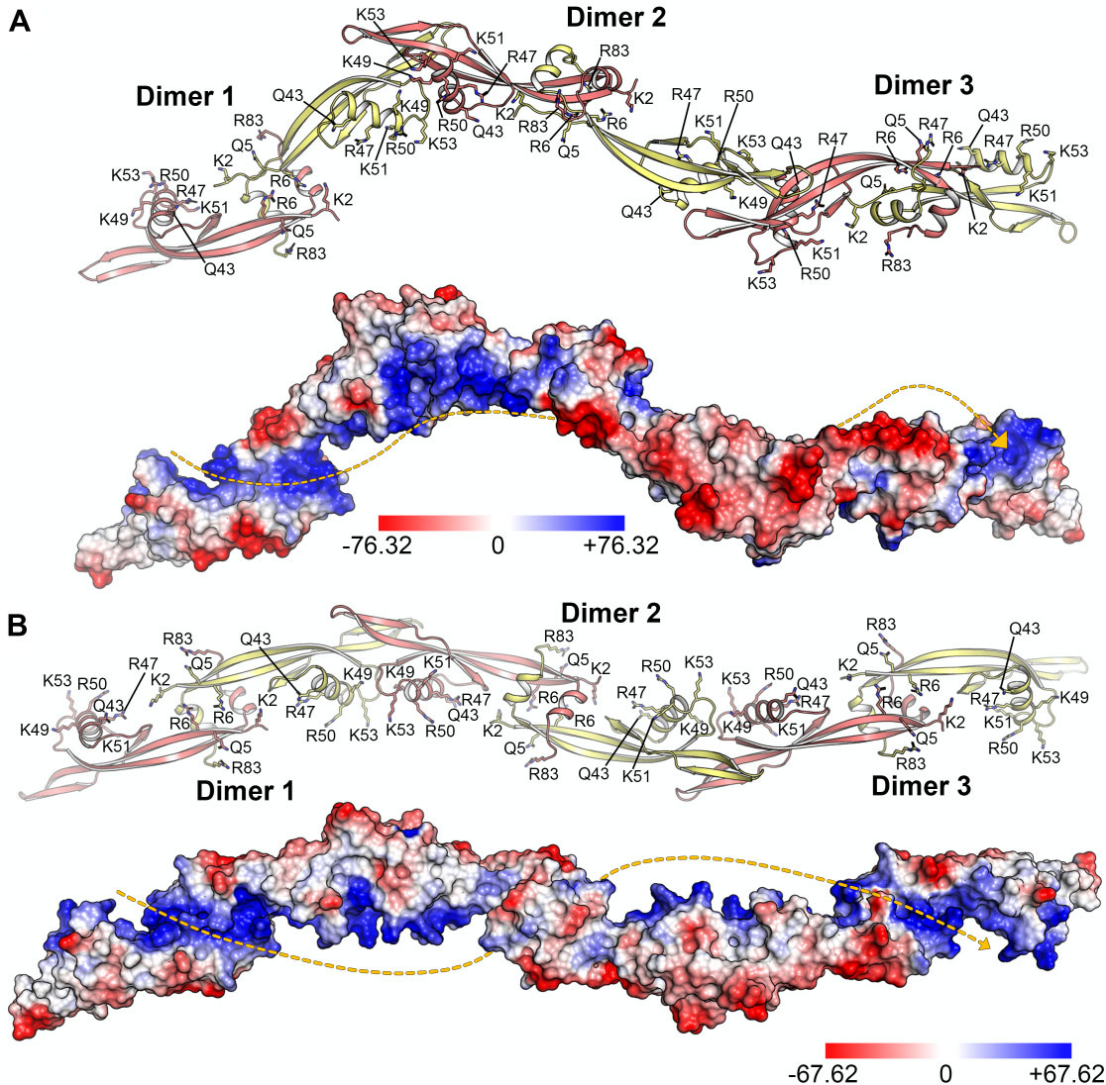
MEP surface on the p6CΔ20 superhelix. (**A**) The p6CΔ20 superhelix as observed in the crystal lattice is depicted in cartoon where each monomer from a dimer is colored in salmon (Monomer 1) and pale yellow (Monomer 2). Residues situated along the basic path are shown in stick representation and labeled. The lower panel shows the MEP surface of the p6CΔ20 oligomer determined by X-ray crystallography. The orientation is consistent with the upper panel, and the color key (blue for positive, red for negative) represents the Poisson-Boltzmann electrostatic-potential surface (color bar range ± 76.32 kT/e). The yellow dashed line highlights the basic path surrounding the p6 oligomer along its longitudinal axis. (**B**) The p6CΔ20 superhelix as predicted by AF-Multimer is presented in a cartoon representation, with each monomer from a dimer colored in salmon (Monomer 1) and pale yellow (Monomer 2). Residues situated along the basic path are shown in stick representation and labeled. The lower panel displays the MEP surface of the p6CΔ20 oligomer predicted by AF-Multimer. The orientation matches that of the upper panel, and the color key (blue for positive, red for negative) represents the Poisson-Boltzmann electrostatic-potential surface (color bar range ± 67.62 kT/e). The yellow dashed line highlights the basic path surrounding the p6 oligomer along its longitudinal axis.

The oligomeric arrangement of p6CΔ20 was further investigated with AF-Multimer (85). The AF-Multimer model strongly aligns with the protein filament structure determined by X-ray crystallography, involving the same interaction regions between monomers (Supplementary Figure S7A). While both structures (experimental *vs* predicted) maintain overall global structural features, there are notable differences worth noting. Firstly, the AF-Multimer prediction shows a more elongated conformation, with four monomers per turn instead of the six observed in the crystallized fiber (Supplementary Figure S7B). Additionally, when considering an equal number of monomers (e.g. six), the AF-Multimer structure displays a greater longitudinal distance along the axis compared to the crystal structure (260 Å versus 230 Å, Supplementary Figure S7C). Conversely, the X-ray oligomer has a larger diameter than the AF-Multimer model (60 Å versus 45 Å, Supplementary Figure S7C). Secondly, in the AF-Multimer model, both the OI and the DI exhibit symmetry, with interacting residues from each monomer being related by a two-fold symmetry axis (Supplementary Figure S7C and D). In the particular case of the OI, the disparity arises because one of the β-hairpins interacts with the β-hairpin of a monomer from the adjacent fiber. Notably, these interaction sites are vital for facilitating the main contacts between the fibers, enabling crystal packing (Supplementary Figure S8). Hence, it is possible that the crystal packing induces a deformation in the superhelix, particularly at the hinge and β-hairpin regions, due to the inherent structural malleability of p6CΔ20. A similar phenomenon has been observed in the crystallographic superhelical structure of the *histone-like* protein H-NS (81). Therefore, the more elongated conformation provided by AF-Multimer may offer a better approximation to the p6 filament in solution. In fact, MD simulations based on the crystal fiber strongly suggest a tendency for the structure to relax from the conformation observed in the crystal and adopt a conformation more similar to that predicted by AF-Multimer (Movie S2). The MEP surface analysis of the p6CΔ20 filament unveils a significant aspect. The positive MEP surface of each dimer, composed of residues K2, Q5, R6, Q43, R47, K49, R50 and R53 (Figure 5A and B and Supplementary Figure S9A), aligns in phase and merges with the positive MEP of the neighboring dimer, forming a continuous basic pathway spanning the entire longitudinal axis of the p6 oligomer. This structural characteristic is observed in both the experimentally determined X-ray crystal structure and the predicted structure from AF-Multimer (Figure 5A and B and Movies S3 and S4). It serves as an ideal framework for accommodating dsDNA, promoting both compaction and gene expression regulation. The presence of basic or polar residues along this pathway correlates with the observed decrease in p6–DNA binding affinity as ionic strength increases (28,87), implying that protein p6–DNA interaction is primarily driven by electrostatic interactions.

### Protein p6–DNA complex

**Figure 6.**
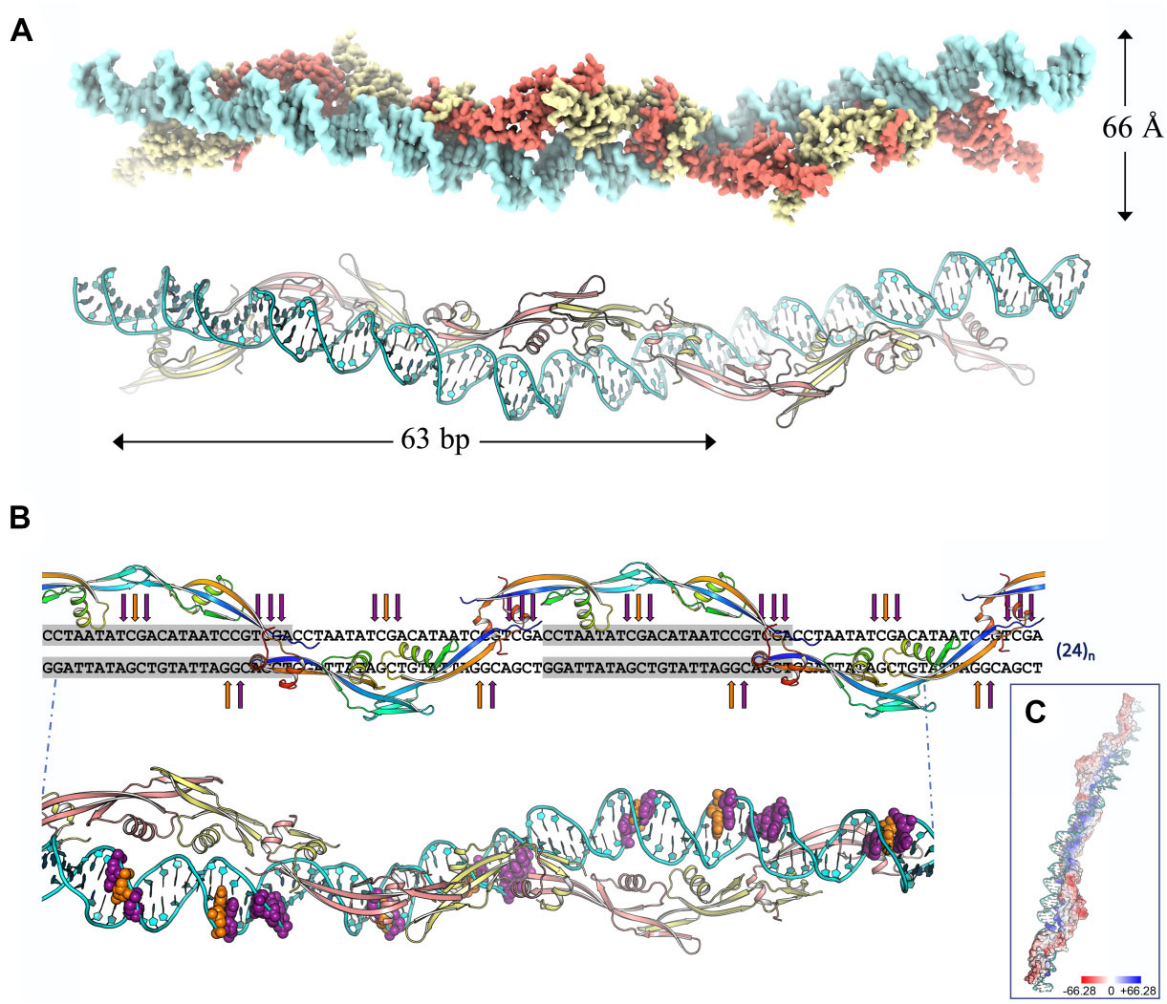
Representative model for the p6CΔ20–DNA complex. (**A**) The proposed complex formed between the right-handed DNA superhelix and a p6CΔ20 filament consisting of three concatemeric dimers. The path of the DNA follows the yellow dashed line shown in Figure 5B. In the upper panel, a surface representation is shown, with each monomer of the p6 dimer colored in salmon (Monomer 1) and pale yellow (Monomer 2). The DNA is represented in cyan. The lower panel presents the same representation as the upper panel, but in cartoon format. (**B**) The concatemeric C24 sequence is shown, highlighting the DNase I hypersensitive sites and the location of the protein p6CΔ20 monomers within the nucleocomplex. The monomers are color-coded in rainbow. The lower panel provides the same representation as the upper panel but in a three-dimensional view in cartoon format. Strong DNase I hypersensitive sites are indicated by orange arrows, while unprotected or medium DNase I hypersensitive sites are indicated by purple arrows. In the lower panel, DNase I hypersensitive sites follow the same color code and are depicted in spheres. (**C**) The MEP surface of the p6CΔ20 oligomer in complex with DNA. The color key (blue for positive, red for negative) represents the MEP surface calculated by solving the Poisson-Boltzmann equation (color bar range ± 66.28 kT/e).

Our attempts to crystallize the p6–DNA complex failed. This could be attributed to the high dynamics and flexibility of the complex, which necessitates a minimum DNA length of ∼100 bp for stable formation (31). Other structural approaches, such as cryo-Electron Microscopy (EM), also present formidable challenges due to two primary limitations. Firstly, the inability to homogeneously isolate the nucleocomplex (due to its dynamic nature) results in heterogeneous mixtures that hamper unambiguous structural determination. Secondly, the complex's integrity is compromised during EM grid preparation, likely attributed to glow-discharge and electrostatic interactions, emphasizing the complex's labile nature. Given these inherent constraints, our goal was to obtain the more accurate representation of the protein fiber conformation in the p6–DNA complex in solution. We aimed to compare the structural information obtained from either AF-Multimer predictions or X-ray crystallography. To achieve this, we utilized the HYDROPRO software (55), which predicts solution properties of macromolecules from their atomic-detailed model (see Materials and methods section). Initially, we conducted analytical ultracentrifugation experiments using SV and SE to quantitatively characterize the DNA binding properties of protein p6CΔ20. These experiments utilized the φ29 left DNA terminal fragment (259 bp-long, referred to as L fragment), which contains the phage's left replication origin (Supplementary Figure S10). The equilibrium gradient of the p6CΔ20–L mixture was well described by a single-species model, yielding a buoyant molecular weight of 133 251 ± 487 Da, corresponding to a 1:22 p6CΔ20–DNA complex. This suggests that the entire L fragment is fully covered with p6CΔ20 molecules (259 bp/12 = 21.58 monomers), consistent with previous findings for the *wt* p6 protein (86). The nucleoprotein complex formed with p6CΔ20 exhibited a sedimentation coefficient of 8.6 ± 0.2 S, notably higher than the DNA alone, with an *s*-value of 6.0 S. Next, we compared the experimentally determined *s*-value for the p6CΔ20–L complex with the predicted value obtained using HYDROPRO. We employed two different structural models for p6CΔ20–L: one derived from the crystal structure of the p6 oligomer and the other based on the AF-Multimer prediction followed by protein docking into DNA and subsequent MD simulations (see below). Both models were subjected to HYDROPRO analysis, resulting in *s*-values of 9.20 S for the X-ray structure and 8.50 S for the AF-Multimer prediction-derived model. Interestingly, the experimentally determined *s*-value (8.6 ± 0.2 S) closely matched the predicted *s*-value for the model derived from AF-Multimer prediction. Therefore, we will solely consider the p6CΔ20 oligomer predicted by AF-Multimer for further exploration of its structural properties in DNA binding. To investigate these properties, we conducted MD simulations to model the p6–DNA complex (Movie S5). For maximizing efficacy, we utilized a concatemeric sequence consisting of repeated copies of a flexible 24 bp sequence (C24) located in the main recognition region of the φ29 DNA left end. The predicted anisotropic bendability properties of this sequence favor protein p6 binding (27) and ensures the generation of a repetitive nucleocomplex (see Materials and methods section). Figure 6A depicts the structure of the p6–DNA complex model, with the repetitive C24 DNA sequence following the longitudinal basic path observed in the p6 filament. Supplementary Figure S9D and E demonstrate the strong correlation between the overall dimensions of the p6 dimer and the expected binding of a 24 bp DNA segment per dimer. In the resulting DNA superhelix, the dimensions are consistent with previously reported values (27), expanding to ∼66 Å in diameter with 63 bp per turn (Figure 6A). In addition, DNase I and hydroxyl radical footprinting analysis revealed the formation of a regular pattern between p6 and DNA, where the protein p6 repeated motif is formed by a protein dimer bound to a 24 bp DNA segment, with the centers of the two monomer binding sites located 12 bp apart (26,28). The DNA–DNase I interaction, which covers over 5 bp, is affected by the width of the minor groove and the bending of the DNA away from the bound enzyme. In our representative p6–DNA complex model, we have located these previously detected DNase I hypersensitivities (Figure 6B), causing widening of the minor groove on the outer side of the curve and facilitating enzyme cleavage. Importantly, these hypersensitivities are situated on the opposite face of the DNA molecule occupied by the protein filament (Figure 6B), enabling the accommodation of the nuclease when p6 is bound (Movie S6). Our model is also supported by the described p6 displacement from DNA by distamycin A (29), a crescent-shape drug that fits snugly into the narrow minor groove of A:T-rich DNA and which presence is incompatible with p6 binding to DNA.

Remarkably, the structure of the p6CΔ20 filament and its proposed mode of interaction with DNA, aligns with other experimental findings. Site-directed mutagenesis studies have highlighted the critical role of residues K2 and R6 in DNA binding *in vitro* and viral DNA synthesis *in vivo* (29,88). These residues are absolutely conserved among the φ29 family of phages (Supplementary Figure S1A and C) and are located in the central region of the basic path (Figure 5A and B). The observed mutant phenotypes likely result from the disruption of direct DNA interactions. Residues K2 and R6, along with other highly conserved residues like R50 and K53 (Supplementary Figure S9A), contribute to the formation of the positive MEP surface. Moreover, a p6 variant with a five amino acid deletion affecting the N-terminus (p6NΔ5) exhibited reduced DNA binding affinity, and when the deletion was extended to 13 amino acids (p6NΔ13), no activity was detected, indicating the involvement of this region in DNA binding (89). Both deletions remove two essential residues (K2 and R6) necessary for DNA interaction (Supplementary Figure S9B and C). Additionally, residue Q5 is also involved in DNA binding, as evidenced by the decreased capacity for complex formation upon Q5A substitution (29), and is appropriately positioned within the basic patch (Supplementary Figure S9A). In contrast, mutagenesis studies have also shown that N14 is not relevant for DNA interaction (29), consistent with its location on the opposite side of the DNA interaction surface in the p6 structure (Supplementary Figure S9E). Furthermore, dimer formation was significantly impaired in p6NΔ5 and completely abolished in p6NΔ13 (80). These deletions affect the N-terminal region of β1, disrupting critical contacts that stabilize the C-terminal part of the antiparallel β4, which is immediately followed by the dimerization helix α2 (Supplementary Figure S3, Supplementary Figure S9B and C). Deletions affecting the carboxy-terminus have also been investigated. The p6CΔ14 mutant remains soluble and stable, while deletions of 23 or more amino acids from the carboxy-terminus render resulting proteins unstable and insoluble (90). Our structural analysis shows that a C-terminal deletion removing 23 amino acids affects the DI, while larger deletions (31 or more residues) completely disrupt dimer formation. (Supplementary Figure S5). As a final observation, it is noteworthy that protein p6 features a unique tryptophan residue at position 46 (W46), which allows for fluorescence quenching studies to investigate DNA-protein binding *in vitro*. The intrinsic fluorescence spectrum of W46 exhibits a maximum at 355 nm (87), indicating its solvent-accessibility. If it were shielded from the solvent, a shift towards 330 nm would be expected. Quenching, induced by the presence of dsDNA, results in a reduction in the fluorescence spectrum area without altering the position of the maximum emission (87). This implies that W46 is not directly involved in the interaction with DNA. Our structural model explains this phenomenon. W46 is exposed to the solvent on one side of the basic path but remains in close proximity to the DNA binding site, playing a fundamental role in orienting the sidechain guanidinium of R50 towards the DNA minor groove in the A, T-rich regions (Supplementary Figure S9E). These regions are precisely the preferred sites for distamycin binding. In summary, the fully atomistic model presented in Figure 6 provides a structural rationale for understanding the observed binding and compaction of dsDNA facilitated by protein p6.

### The C-terminal tail of protein p6 plays a crucial role in dimerization and modulates its DNA-binding properties

**Figure 7.**
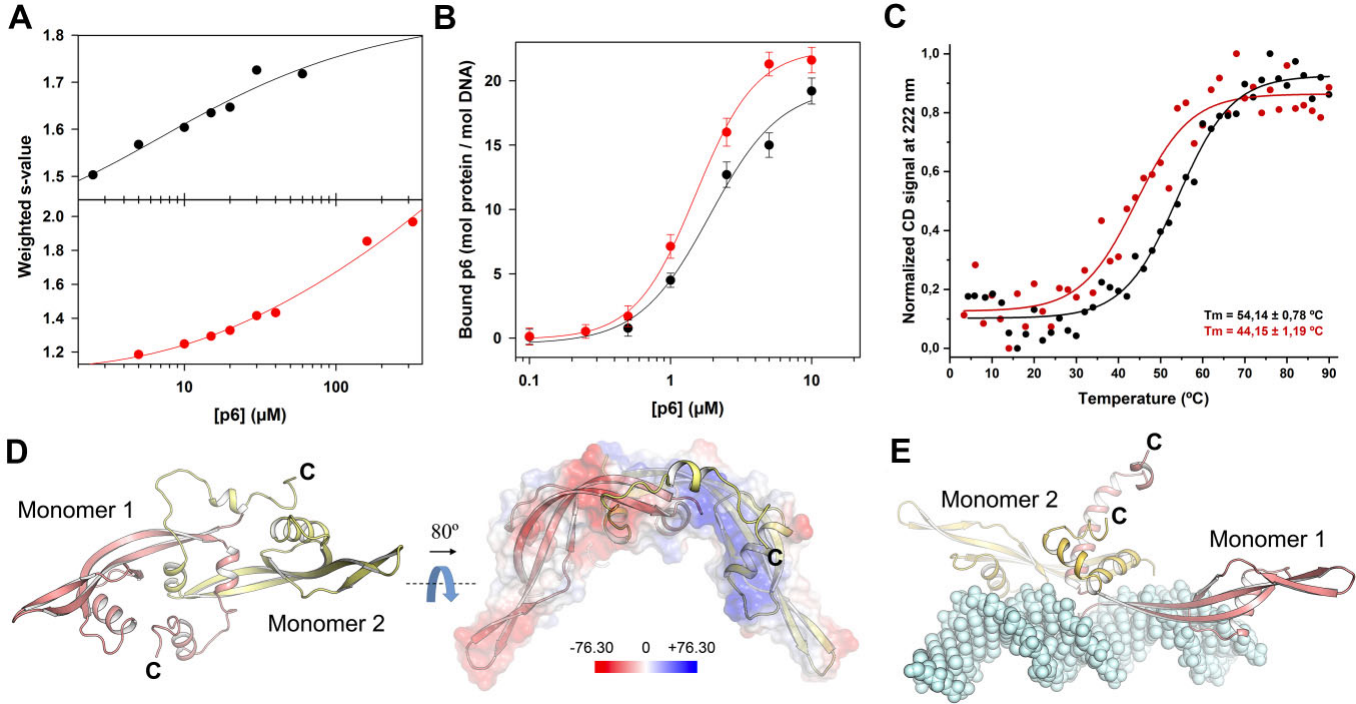
Influence of the protein p6 acidic tail in protein self-association and DNA binding. (**A**) Self-association isotherms (in the range 2.5–320 μM) built from weight-average sedimentation coefficients of *wt* p6 (black circles) and p6CΔ20 (red circles) and analyzed through a monomer-dimer self-association binding model as implemented in SEDPHAT (53). (**B**) Binding isotherms for the interaction of 0.1 μM DNA**–**L with increasing concentrations of *wt* p6 (black circles) and p6CΔ20 (red circles). The solid curves represent the best fit of the three-parameters Hill equation (Equation 2) to the SE experimental data (for details, see Materials and methods). (**C**) The normalized CD signal at 222 nm for the *wt* p6 (black circles) and p6CΔ20 (red circles) against temperature is shown. The denaturation curves clearly showed the role of the acidic tail in the thermodynamic stability increase of p6 protein (*T*_m_ = 54ºC for *wt* p6 versus *T*_m_ = 44ºC in p6CΔ20). The buffer contained 20 mM NaPO_4_ at pH 7.4 and 50 mM NaF. (**D**) The dimeric arrangement of the *w*t protein p6 was investigated by means of MD simulations. A cartoon representation of the protein dimer is shown, with Monomer 1 colored salmon and Monomer 2 colored pale yellow. In the right panel, the dimer is depicted after an 80º rotation around the indicated axis. The semitransparent MEP surface highlights the interaction between the C-terminal tail of Monomer 2 and its corresponding basic patch (shown in blue). (**E**) MD simulations were performed for the *wt* p6 dimer in complex with DNA. A representative frame is shown in which both C-terminal tails are depicted. Although their positions fluctuate during the dynamics, they do not interact with the DNA. C: carboxy-terminus.

The acidic nature of the C-terminus of p6, characterized by a high content of Asp and Glu residues (Supplementary Figure S1A), is a crucial factor regulating the protein's DNA affinity. Previous reports showed that the presence of this region decreases the protein's DNA affinity (80,90), while its absence leads to higher viral DNA replication yields (90). We aimed to investigate the functional significance of the acidic tail in (i) self-association, (ii) DNA binding and (iii) protein stability. First, we conducted SV experiments comparing the dimerization behavior of p6CΔ20 with that of the *wt* p6 at different concentrations. The peaks from the *c*(*s*) distributions obtained were integrated to build the corresponding weight-average sedimentation coefficients isotherms. As shown in Figure 7A, removal of the last 20 residues reduced the dimerization ability of p6, increasing the dimerization constant (K_2_) from 11.4 μM (for the *wt* p6) to 47.0 μM (for the p6CΔ20 mutant). Further deletion involving the α2 helix (p6CΔ31) completely abolished dimerization (not shown). Second, we studied the binding of p6CΔ20 to DNA using SE, specifically examining the binding of increasing protein concentrations to a fixed amount (0.1 μM) of the L fragment (see Materials and methods). At a concentration of 10 μM, the observed increase in buoyant molecular weight for both *wt* p6 and p6CΔ20 corresponded to 19.2 and 21.6 protein monomers, respectively, consistent with previously reported data for *wt* p6 (86). Considering that the maximum increase in buoyant mass at the highest protein concentration corresponds to the binding of 21.58 protein monomers [259 bp/12 = 21.58, as reported previously (86)], we can propose a binding model accompanied by its corresponding macroscopic dissociation constant (*K*_d_). This model takes into account the experimental binding isotherms shown in Figure 7B. These experiments revealed a lower *K*_d_ of 1.47 ± 0.1 μM for the p6CΔ20 mutant compared to the *K*_d_ of 2.46 ± 0.3 μM for the *wt* p6. This quantitative result indicates that the absence of the acidic tail enhances protein DNA-binding, acting as a negative regulator of protein binding to DNA. Third, CD thermal denaturation experiments demonstrated that the acidic tail contributes to protein stability, resulting in an observed decrease in Tm of around 10ºC, compared to the *wt* p6 (Figure 7C). These results highlight its importance in maintaining protein integrity.

To elucidate the functional implications of the C-terminal tail in protein self-association, stability, and its negative modulation of DNA binding, we employed MD simulations. Specifically, we focus on a *wt* p6 dimer obtained from AF-Multimer to unravel the underlying mechanisms (Materials and methods). The MD simulations indicated a potential interaction between the acidic tail and the basic patch of its corresponding monomer within the dimer, consistently supporting the stable maintenance of this electrostatic interaction over time. In the dimer, this interaction would occur after the connection facilitated through the α2 helix of each monomer, contributing to its overall stabilization (Figure 7D). Furthermore, the MD simulations revealed a propensity of the C-terminal acidic tail to adopt a helical conformation, albeit without long-term stability. This interaction model provides a structural understanding of the acidic tail's role in modulating DNA binding. Accordingly, we propose that this terminal tail functions as an electrostatic shield that, in the absence of DNA, interacts with the basic patch of its corresponding monomer. This would effectively shield the positively charged DNA-binding site from binding to randomly charged surfaces and sensing the presence of viral DNA. In turn, MD simulations of the p6 nucleocomplex revealed that the acidic tail does not engage in direct DNA interactions (Figure 7E).

### Comparative structural analysis of protein p6 within the *Salasvirus* genus of phages

**Figure 8.**
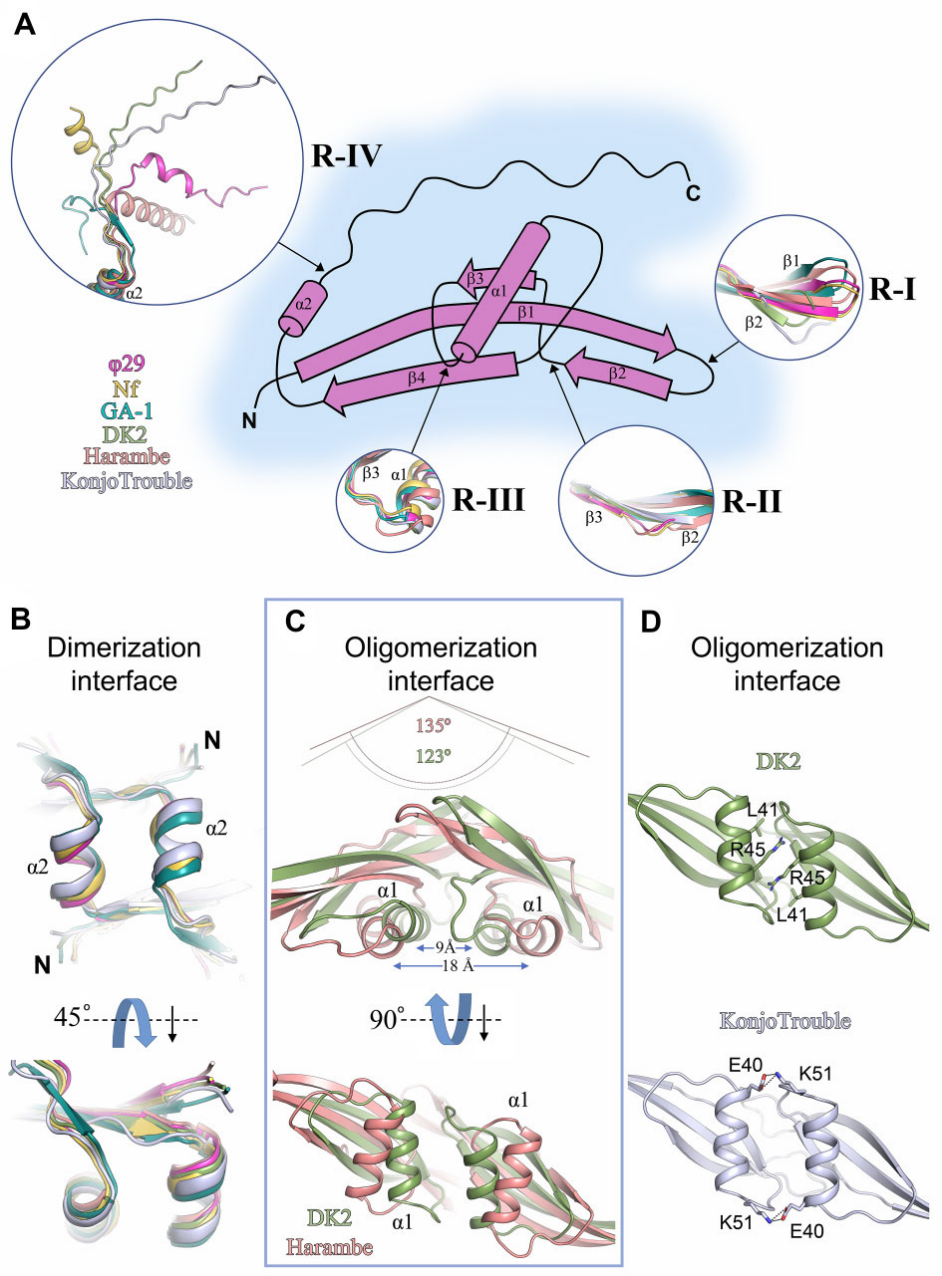
Common structural motif in the p6 monomer and regions of variability in phages from the *Salasvirus* genus. AF-Multimer predictions of the protein p6 monomer from the specified phages. Differences were represented in cartoon and aligned, with the variations overlaid onto the conserved topological diagram of the φ29 protein p6 (see main text for details).

To assess the evolutionary conservation or divergence of p6 structural and functional motifs, we performed a comparative structural analysis on protein p6 from different species within the *Salasvirus* genus of phages. For this, we ran AF-Multimer predictions for all the 35 available sequences corresponding to *gene 6* in identified φ29 relatives. Our analysis revealed that despite the lack of sequence conservation, protein p6 maintains a consistent global fold (with a *rmsd* value ranging from 0.77 to 2.05 Å, using φ29 protein p6 as reference) and consistent DNA binding properties across all phages. This uniformity results in the presence of a helically arranged positive MEP surface along the protein fiber's longitudinal axis (Supplementary Figure S11). This also means that dimerization and oligomerization occur similarly in all φ29 relatives, involving comparable interfaces. However, there are distinctions among these phages that emphasize their unique characteristics. Specifically, we have identified four regions (R-I, R-II, R-III and R-IV; Figure 8) within the p6 monomer that exhibit significant structural variability among species. For further examination, we selected six representative phages (φ29, Nf, GA-1, DK2, Harambe and KonjoTrouble) where structural differences are more pronounced (Figure 8 and Supplementary Figure S11). These chosen phages exemplify the divergence observed in the phylogenetic tree shown in Supplementary Figure S1B, where phages grouped in the same branch of the tree share a similar structure that locally differs from those grouped in other branches. The first region identified, located at the β-hairpin (R-I, residues 17–29 in φ29), displays structural plasticity and variable length. However, this region consistently encompasses multiple hydrophobic residues crucial for oligomerization. At the OI, the angle between the two tightly packed β-hairpins varies depending on the phage species, ranging from 123º (phage DK2) to 135º (phage Harambe), as observed in the two most extreme cases (Supplementary Figure S12A). Furthermore, the distance between the α1 helices also exhibits variability, ranging from 9 Å (phage DK2) to 18 Å (phage Harambe) (Supplementary Figure S12A). These structural distinctions significantly influence the overall dimensions and arrangement of the protein filament (Supplementary Figure S11). Additionally, helices α1 may approach each other at the OI either through hydrophobic packing of residues from both helices (phage DK2, Supplementary Figure S12B) or due to the presence of peripheral salt bridges (phage KonjoTrouble). In contrast, when the p6 structures are superimposed through their DI, it becomes apparent that the conformation of this region remains remarkably consistent across all phage species (Supplementary Figure S13B). This region harbors two hydrophobic residues (Y69 and F76, Supplementary Figure S13A) that are strictly conserved, except in phages PumA1 and Karezy where the Y is replaced by a F (Supplementary Figure S1A).

These residues form a closely packed hydrophobic patch enriched with additional peripheral hydrophobic residues (Supplementary Figure S13A and B). This hydrophobic patch likely plays a crucial role in stabilizing the DI, ensuring a uniform conformation among all p6 proteins in the φ29 relatives. The second region (R-II, residues 29–33 in φ29) encompasses the linker between β2 and β3, typically including a proline residue (phages φ29, Nf and Harambe). However, in its absence, β2 and β3 fuse into a single β structure (phages GA-1, DK2 and KonjoTrouble). The third region (R-III, residues 36–41 in φ29) exhibits substantial conformational heterogeneity, primarily affecting the linker between β3 and α1. It may feature an insertion (phages Nf and Harambe) or the deletion of several residues (phage GA-1). The fourth region (R-IV) concerns the C-terminal part that includes the disordered acidic tail. Its length varies among different phages and may contain an additional β structure (as observed in GA-1), contributing to dimerization. In several cases (phages φ29, Nf and Harambe), this region is predicted to form an α-helix with varying length. Here, we have determined that this region is involved in dimerization and negatively affects DNA binding.

The superhelical arrangement of protein p6 filaments holds significant functional implications that influence the stability, the mechanical properties and the biological activities of the filament. Given that the predicted p6 filaments of various φ29-like phages display distinct helical characteristics, it is tempting to draw an inverse relationship between this structural property and the DNA supercoiling dependency of each viral system. Notably, the protein p6 of bacteriophage GA-1 forms a more compact filament with a more pronounced helical structure compared to other φ29-like phages (Supplementary Figure S11). Consistent with this notion, the DNA binding behavior of GA-1 protein p6 exhibits little dependence on DNA supercoiling, in contrast to the orthologous protein of phage φ29 (39). This lower dependency on supercoiling of GA-1 p6 with respect to φ29 p6 also reflects the different structure of the nucleocomplex formed by each protein. In the case of φ29, p6 monomers bind to DNA every 12 bp (26), while GA-1 p6 monomers bind to DNA every 11 bp (38).

### Concluding remarks

In this work, we have shown that protein p6 showcases a remarkable evolutionary design that encompasses a folded core as its foundation, upon which, two structural regions play crucial roles in dimerization and oligomerization. Whereas the folded core of protein p6 forms the stable scaffold, providing the structural integrity and stability necessary for its function, the two adjacent regions are pivotal for its functional versatility. These regions, characterized by their dynamic nature and conformational flexibility, adopt different conformations and orientations to fulfill their specific roles. One of these regions is dedicated to dimerization, enabling p6 to form a stable complex with another p6 molecule, primary through hydrophobic interactions involving helix α2 and the long C-terminal acidic tail. The hinge region preceding α2 allows the folded core to adopt diverse orientations, orchestrating the formation of functional dimeric units essential for vital processes like DNA replication and transcription during the viral life cycle. The other relevant region of p6 involves the β-hairpin, which plays a role in oligomerization, where multiple p6 dimers concatenate to form larger assemblies or filaments through the hydrophobic stacking of their respective β-hairpins.

Our detailed analysis of protein p6 structures and molecular models, along with its intricate interplay with DNA, provides a comprehensive description of how the protein interacts with DNA, in agreement with most experimental data reported. We have identified only two minor discrepancies that can be reinterpreted in light of our findings. Firstly, the N-terminal region of protein p6, previously thought to form a 25-residue α-helix serving as a DNA-binding domain recognizing the minor groove (29), is revealed to adopt a β-strand structure. Despite this difference, our results highlight that this region is still critical for DNA interaction through the minor groove. Secondly, previous studies estimated DNA compaction induced by p6 in the nucleocomplex to be 4 to 6-fold using EM (27,31). However, our solution-based measurements indicate a more moderate DNA compaction induced by p6, implying that EM analyses may have artificially overestimated compaction. This overestimation could be attributed to the sample manipulation required for its visualization under the microscope (for instance, the intramolecular DNA condensation during specimen dehydration). Despite these discrepancies, the remaining parameters describing the nucleocomplex structure (primary encompassing the number of nucleotides covered by each protein monomer, number of bps per DNA turn, with of the nucleocomplex and results from mutagenesis studies) align well with our findings, demonstrating a significant agreement between our results and the proposed molecular framework.

In summary, our results provide valuable insights into the structural aspects of p6 and its interaction with dsDNA, enhancing our understanding of the nucleoprotein complex formation during viral replication. The self-association of p6 into high-order macrostructures, which is concentration-dependent, showcases a finely tuned mechanism responsive to the environment. This mechanism enables p6 to fulfill two important functions: binding and compacting DNA, while also controlling access to the protein machinery involved in gene transcription and DNA replication. Positive DNA supercoiling, facilitated by p6’s binding to internal high affinity DNA stretches, must lead to the generation of accumulated tension. This torsional stress must be effectively dissipated through compensatory changes, likely serving as the driving force for strand separation at the adjacent replication origins, particularly in A:T-rich regions. This should be possible because the linear viral genome, although not covalently closed, is topologically constrained (91), most probably due to membrane attachment (92), presumably through the TPs, which have intrinsic affinity for the membrane (93). Consequently, the precise positioning of protein p6 on viral DNA appears to play a role in opening replication origins, enabling DNA to adopt a suitable configuration for the viral polymerase-mediated initiation of replication, and would be particularly relevant under physiological salt concentration (24). Further investigations are needed to comprehensively elucidate the molecular mechanisms governing p6’s role in viral replication and transcriptional regulation.

## Supplementary Material

gkae041_Supplemental_Files

## Data Availability

Atomic coordinates for the reported crystallographic p6 structures have been deposited at the Protein Data Bank under accession numbers 8PW2 (p6CΔ31) and 8PW4 (p6CΔ20).
