# Supplementary material for: Flexible structural arrangement and DNA-binding properties of protein p6 from Bacillus subtillis phage φ29
Source: Nucleic Acids Res. 2024 Jan 28;52(4):2045–65. doi: 10.1093/nar/gkae041 (PMC10899789; doi:10.1093/nar/gkae041)
Supplement: gkae041_Supplemental_Files [file gkae041_supplemental_files.zip › Supplementary Figures and Movies S1-6 legends.pdf]

“Flexible structural arrangement and DNA-binding properties of protein p6 from Bacillus subtillis phage φ29”

Supplementary Figures:

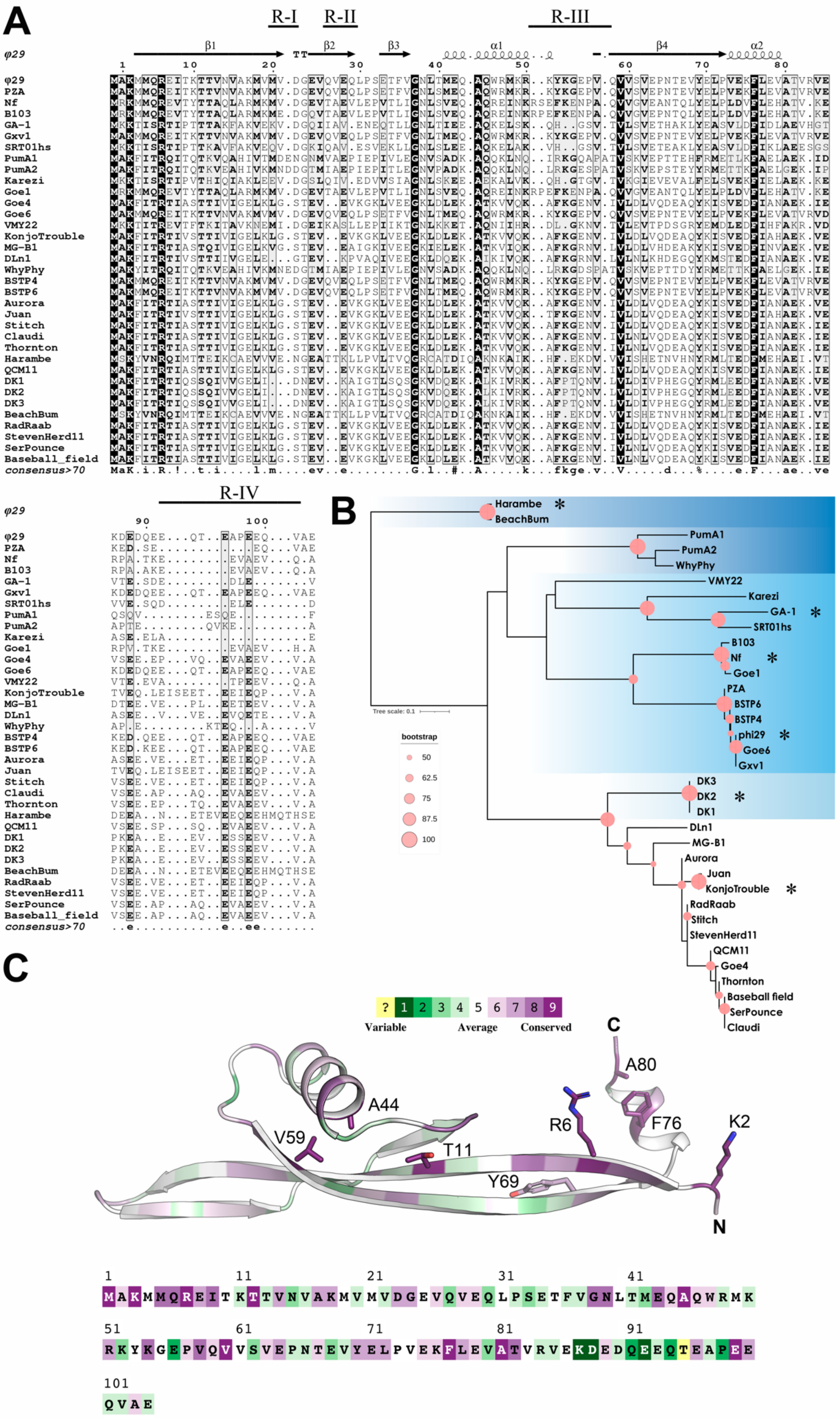
A
R-I
R-II
R-III
φ29
β1
β2
β3
α1
β4
α2
φ29
PZA
Nf
B103
GA-1
Gxv1
SRT01hs
PumA1
PumA2
Karezi
Goe1
Goe4
Goe6
VMY22
KonjoTrouble
MG-B1
DLn1
WhyPhy
BSTP4
BSTP6
Aurora
Juan
Stitch
Claudi
Thornton
Harambe
QCM11
DK1
DK2
DK3
BeachBum
RadRaab
StevenHerd11
SerPounce
Baseball_field
consensus>70
R-IV
B
Harambe
BeachBum
PumA1
PumA2
WhyPhy
VMY22
Karezi
GA-1
SRT01hs
B103
Nf
Goe1
PZA
BSTP6
BSTP4
phi29
Goe6
Gxv1
DK3
DK2
DK1
DLn1
MG-B1
Aurora
Juan
KonjoTrouble
RadRaab
Stitch
StevenHerd11
QCM11
Goe4
Thornton
Baseball field
SerPounce
Claudi
Tree scale: 0.1
bootstrap
50
62.5
75
87.5
100
C
? 1 2 3 4 5 6 7 8 9
Variable
Average
Conserved
C
N
A80
A44
R6
F76
K2
V59
T11
Y69
1
11
21
31
41
M A K M M Q R E I T K T T V N V A K M V M V D G E V Q V E Q L P S E T F V G N L T M E Q A Q W R M K
51
61
71
81
91
R K Y K G E P V Q V V S V E P N T E V Y E L P V E K F L E V A T V R V E K D E D Q E E Q T E A P E E
101
Q V A E

Supplementary Fig. 1. (**A**) Protein p6 family sequence alignment produced by T-COFFEE (1) and drawn with ESPript (2). Identities are boxed in black. Similarities are boxed in gray according to physico-chemical properties. Secondary structure elements have been calculated from the AF2 structure prediction of φ29 protein p6 using the program DSSP (3). They are displayed on the top of sequence blocks. Alpha helices are represented by squiggles labelled α. Strands are represented by arrows. Sequence numbering corresponds to φ29 protein p6 sequence. According to AF2 predictions, regions R-I, R-II, R-III and R-IV cluster significant structural variability (see Discussion for details). (**B**) ML phylogenetic hypothesis for p6 viral proteins. Only bootstrap values over 50 are mapped on the nodes. Phylogenetic analysis was performed on a MAFFT alignment of p6 family of proteins (4) using BLOSUM62 model obtained in jModelTest (5). Maximum likelihood analysis was performed with RAxML v2.0 (6) with 100 bootstrap values mapped on the consensus tree and visualized using iToL (7). Chosen phages that are representative of the divergence observed in the phylogenetic tree are labeled with * (See Discussion for details). (**C**) A ConSurf (8) analysis for the AF2 predicted structure of full-length φ29 protein p6. The disordered acidic C-terminal tail has been omitted for clarity. The residues in the 3D cartoon structure as well as in the primary sequence are colored by their conservation grades using the nine-grade color-coding bar, with turquoise-through-maroon indicating variable-through-conserved. Yellow colored residues in the sequence panel denote regions that were assigned conservation level with low confidence due to insufficient data and hence have been excluded from the analysis. Highly conserved residues are indicated and depicted as sticks. N, amino-terminus; C, carboxy-terminus. The sequence of φ29 protein p6 is annotated below showing the conservation quality of amino acid residues. The ConSurf analysis was carried out using the multiple sequence alignment given in A.

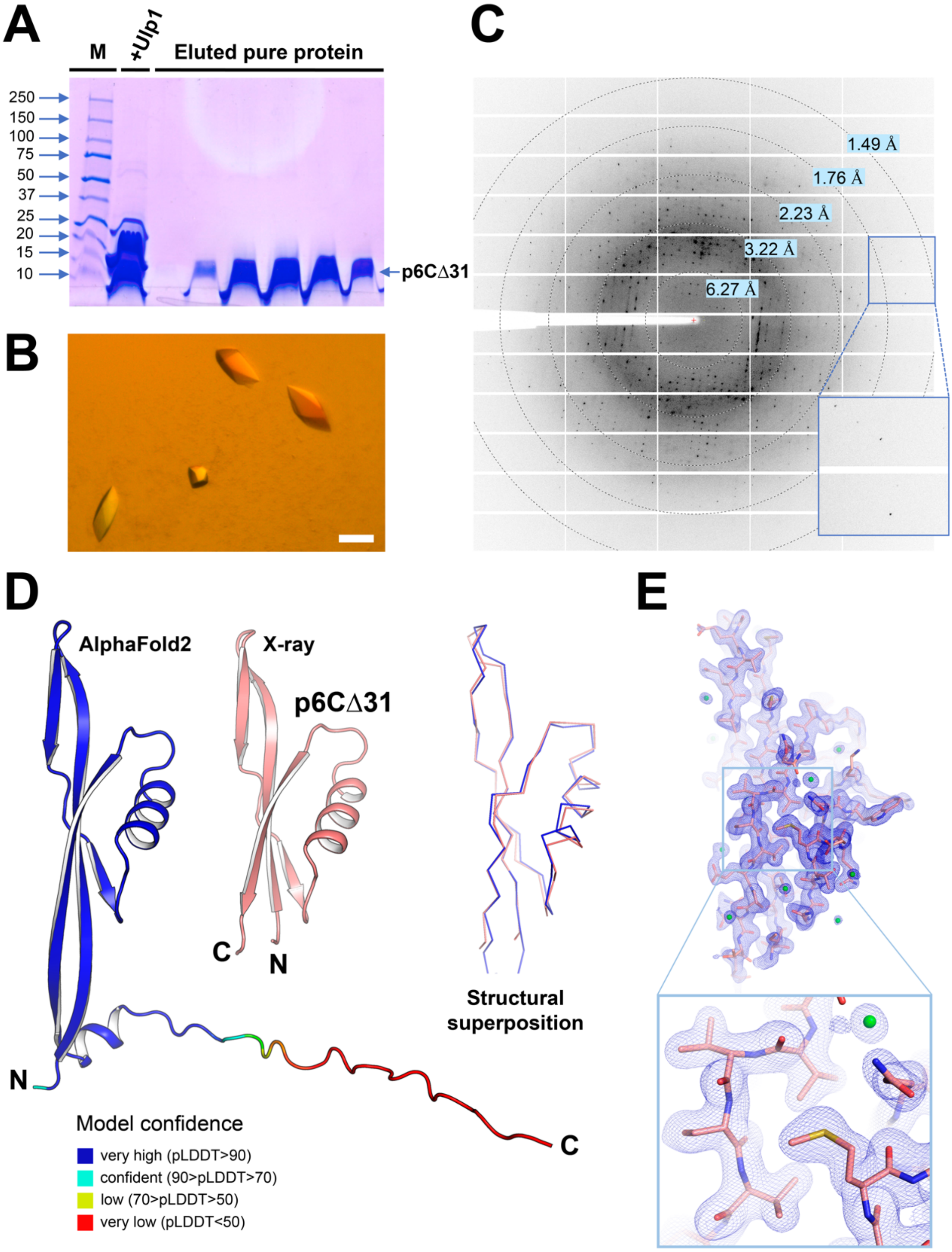

Supplementary Fig. 2. **Protein p6CΔ31 purification, crystallization, data collection and three-dimensional structure.** (**A**) SDS-PAGE (4-15%) analysis of the eluted pure p6CΔ31 after His-tagged-SUMO removal with Ulp1 protease. Weight-molecular markers (kDa) are indicated for the left lane. (**B**) p6CΔ31 crystals obtained in 0.1 M Bis–Tris Propane pH 7.0 and 1.3 M di-Ammonium Tartrate. Scale bar represents 0.3 mm. (**C**) Diffraction image of p6CΔ31 crystal collected in beamline XALOC at the ALBA synchrotron using a Pilatus 6M detector. Resolution rings are indicated with dashed lines. The close-up view shows an example of reflections around 1.6 Å resolution and beyond. (**D**) Cartoon representation of the predicted AF2 structure of the full-length protein p6 (color-coded by model confidence) and the experimental p6CΔ31 crystallographic structure (salmon). Right panel shows how good is the structural superposition between both structures, depicted as Cα trace (*rmsd* of 0.664 Å across 53 pruned atom pairs). N, amino-terminus; C, carboxy-terminus. (**E**) Electron-density map (2Fo-Fc map contoured at 1σ) for the ~1.6 Å resolution structure of p6CΔ31 monomer. The boxed region shows a close-up view of the map in

which electron densities at the above-mentioned resolution can be appreciated. Water molecules are depicted as green spheres.

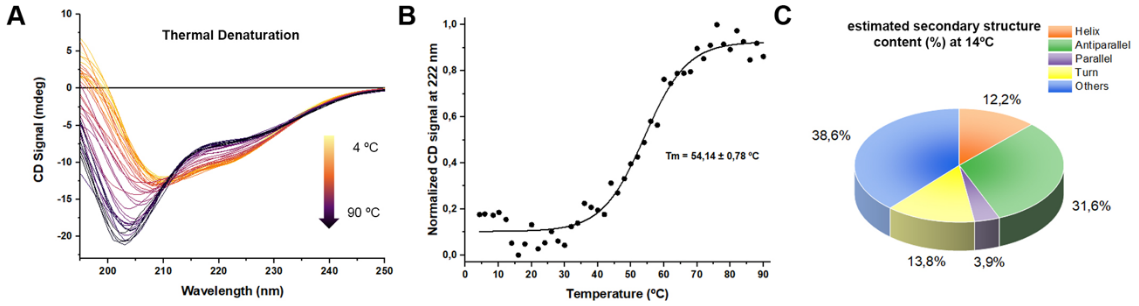

Supplementary Fig. 3. **CD spectroscopy measurements performed on the p6 *wt* protein. A**) Thermal denaturation CD spectra for the *wt* protein at 14.5 µM. **B**) The mid-denaturation temperature (Tm) of the *wt* protein was calculated by analyzing the CD data at 222 nm during the unfolding process using Boltzmann fitting implemented in Origin Software. **C**) The estimation of the secondary structure percentage for the *wt* protein at 14ºC was determined using the BestSel tool. Structural predictions were obtained for eight different structural elements: *Helix 1* (the central regular segment of α-helix), *Helix 2* (distorted region at the end of α-helices), *Anti 1* (left-hand twisted antiparallel β-sheet), *Anti 2* (relaxed, slightly right-hand twisted antiparallel β-sheet), *Anti 3* (right-hand twisted antiparallel β-sheet), *Parallel β-sheet*, *Turn* [as defined by DSSP (3)], and *Others* (including 3_{10}-helix, π-helix, β-bridge, bend, loop/irregular, and invisible regions of the structure). These elements were categorized into five groups: Helix (comprising Helix 1 and 2), Antiparallel (including Anti 1, 2, and 3), Parallel β-sheet, Turns, and Others.

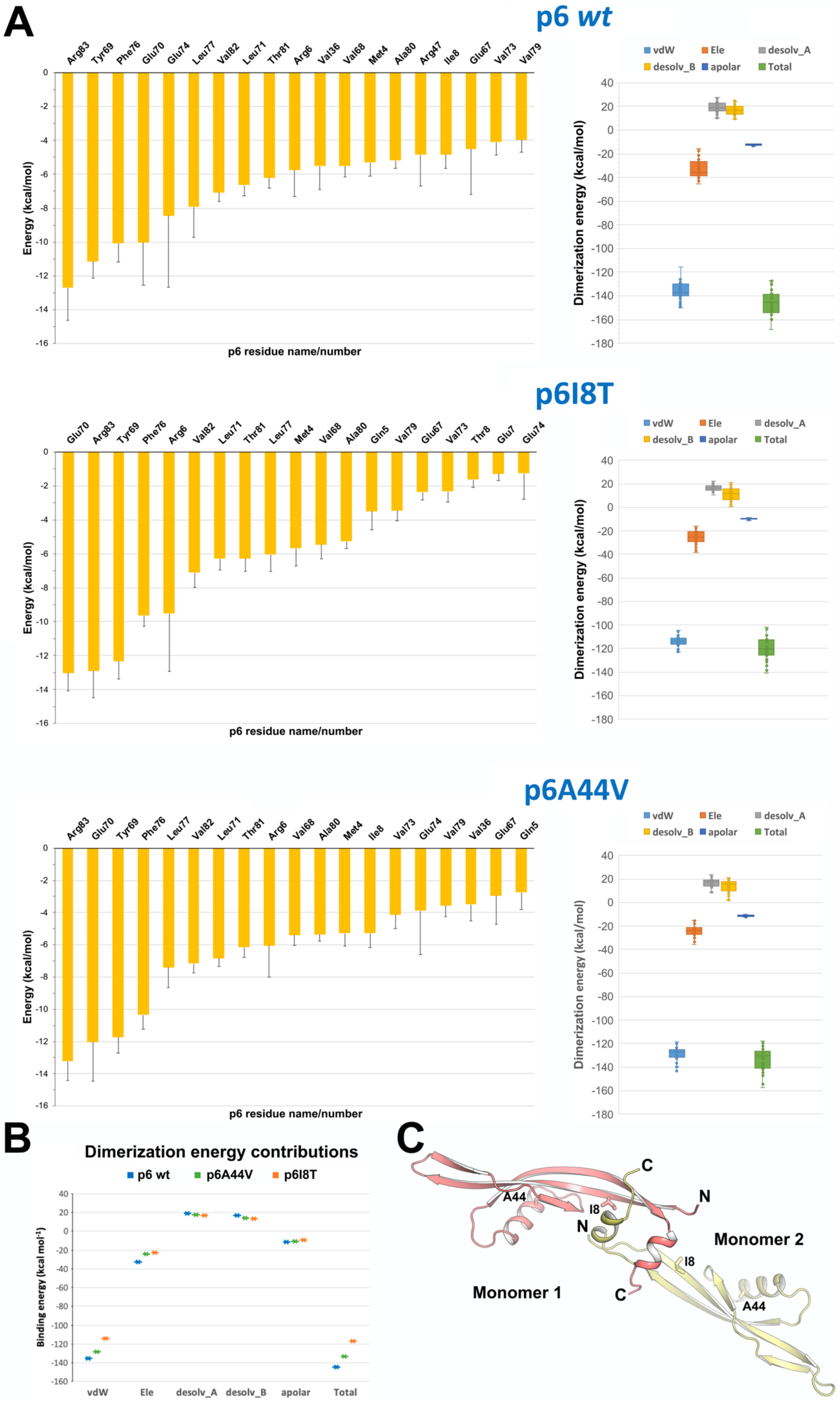
A
p6 wt
Arg83 Tyr69 Phe76 Glu70 Glu74 Leu77 Val82 Leu71 Thr81 Arg6 Val36 Val68 Met4 Ala80 Arg47 Ile8 Glu67 Val73 Val79
Energy (kcal/mol)
p6 residue name/number
vdW Ele desolv_A desolv_B apolar Total
Dimerization energy (kcal/mol)
p6I8T
Glu70 Arg83 Tyr69 Phe76 Arg6 Val82 Leu71 Thr81 Leu77 Met4 Val68 Ala80 Gln5 Val79 Glu67 Val73 Thr8 Glu7 Glu74
Energy (kcal/mol)
p6 residue name/number
vdW Ele desolv_A desolv_B apolar Total
Dimerization energy (kcal/mol)
p6A44V
Arg83 Glu70 Tyr69 Phe76 Leu77 Val82 Leu71 Thr81 Arg6 Val68 Ala80 Met4 Ile8 Val73 Glu74 Val79 Val36 Glu67 Gln5
Energy (kcal/mol)
p6 residue name/number
vdW Ele desolv_A desolv_B apolar Total
Dimerization energy (kcal/mol)
B
Dimerization energy contributions
p6 wt p6A44V p6I8T
Binding energy (kcal mol-1)
vdW Ele desolv_A desolv_B apolar Total
C
A44
I8
N
C
Monomer 1
Monomer 2

Supplementary Figure 4. **Solvent-corrected energy contributions (kcal mol^{-1}) to p6 dimerization.** (**A**) Left panel, average contributions (± standard error) of individual p6 residues (for simplicity, those below a threshold of 1.0 are not displayed) to the overall dimerization energy. Right panels, box-and-whisker plots of the calculated component contributions (van der Waals (light blue), electrostatic (orange), ligand desolvation (grey), receptor desolvation (yellow), and apolar (dark blue) to the total interaction energies (green, kcal mol^{-1}). The averages were calculated from a conformational ensemble made up of 40 snapshots taken every 5 ns from the post-equilibrated 1–200 ns interval of the MD trajectories and then cooled down to 273 K and energy minimized. (**B**) Comparison of the overall calculated total and component contributions (van der Waals (light blue), electrostatic (orange), ligand desolvation (grey), receptor desolvation (yellow), and apolar (dark blue) to the dimerization energies (green, kcal mol^{-1}) of wild-type and variant p6 proteins. (**C**) Cartoon representation of the p6CΔ20 dimer showing the positions of amino acids 8 and 44, represented as capped sticks. Monomer 1 of the dimer is colored salmon, and Monomer 2 is colored pale yellow.

Deletion mutant	Location in the 3D structure	Dimerization	dsDNA binding	Initiation complex	Viral DNA replication	Reference
**p6NΔ5**	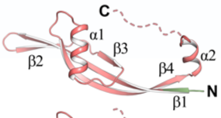	Very reduced	Very reduced	Very reduced	Very reduced	(10)
**p6NΔ13**	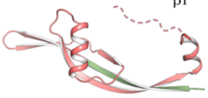	ND	ND	ND	ND	(10)
**p6CΔ14**	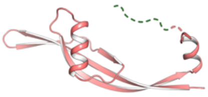	Able to dimerize	Better than *wt*	Better than *wt*	Better than *wt*	(11)
**p6CΔ16**	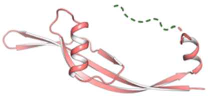	Able to dimerize	Better than *wt*	Better than *wt*	Better than *wt*	(9, 11)
**p6CΔ20**	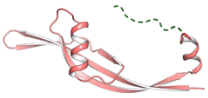	Able to dimerize	Better than *wt*	NA	NA	This work
**p6CΔ23**	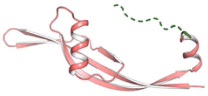	NA	Reduced	Reduced	Reduced	(11)
**p6CΔ31**	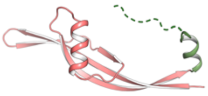	Unable to dimerize	ND	NA	NA	This work
**p6CΔ37**	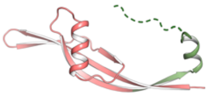	NA	Very reduced	Very reduced	Very reduced	(11)
**p6CΔ38**	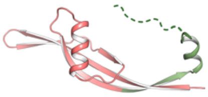	NA	Very reduced	Very reduced	Very reduced	(11)
**p6CΔ39**	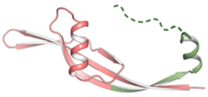	NA	Very reduced	Very reduced	ND	(11)
**p6CΔ44**	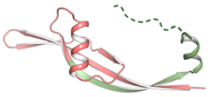	NA	ND	ND	ND	(11)

Supplementary Fig. 5. **Location and phenotype of reported N- and C-terminal deletions of protein p6** (9–11). In the following Table/Figure, a comprehensive summary of the position and observed phenotypic effects associated with various N- and C-terminal deletions that have been reported to affect protein p6 is presented. The deletion of specific residues in each p6 mutant is indicated in the left column. The corresponding locations and impact of these deletions are depicted in green color on the three-dimensional structure of p6CΔ20 (Monomer 1), represented as a salmon-colored cartoon. The phenotypes associated to each deletion is shown in the right columns. The dashed line represents the acidic C-terminal tail. NA, not assayed; ND, not detected. N, amino-terminus; C, carboxy-terminus.

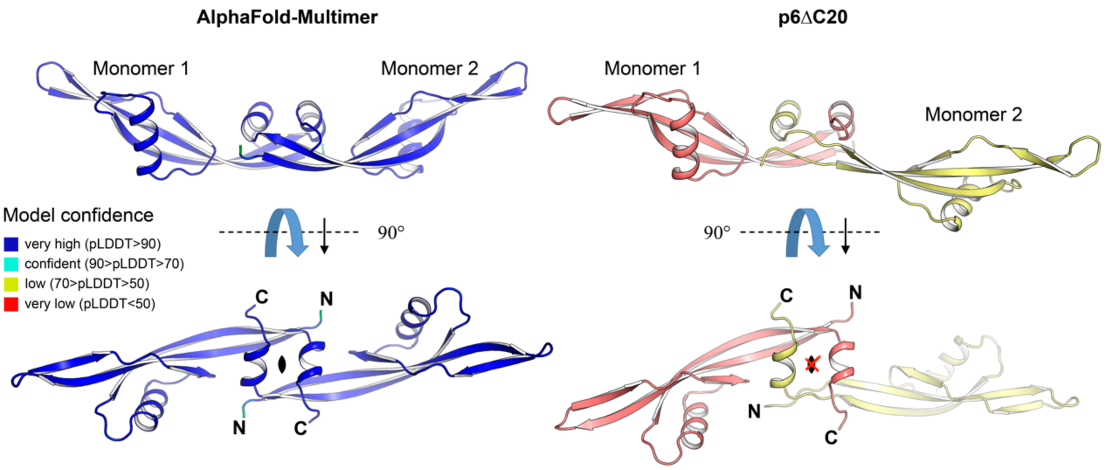

Supplementary Fig. 6. AF-Multimer structure prediction of the p6CΔ20 dimer and comparison with the p6CΔ20 dimer structure obtained by X-ray crystallography. Left panel shows a cartoon representation of the predicted AF-Multimer structure of the p6CΔ20 dimer (color-coded by model confidence). Right panel shows a view of protein p6CΔ20 dimer obtained by X-ray crystallography following the same orientation of the structures shown on the left. Monomer 1 of the p6CΔ20 dimer is colored salmon, and Monomer 2 is colored pale yellow. In both panels, the structures are displayed in two orientations at 90° of each other. The presence of a two-fold symmetry axis is indicated. N, amino-terminus; C, carboxy-terminus.

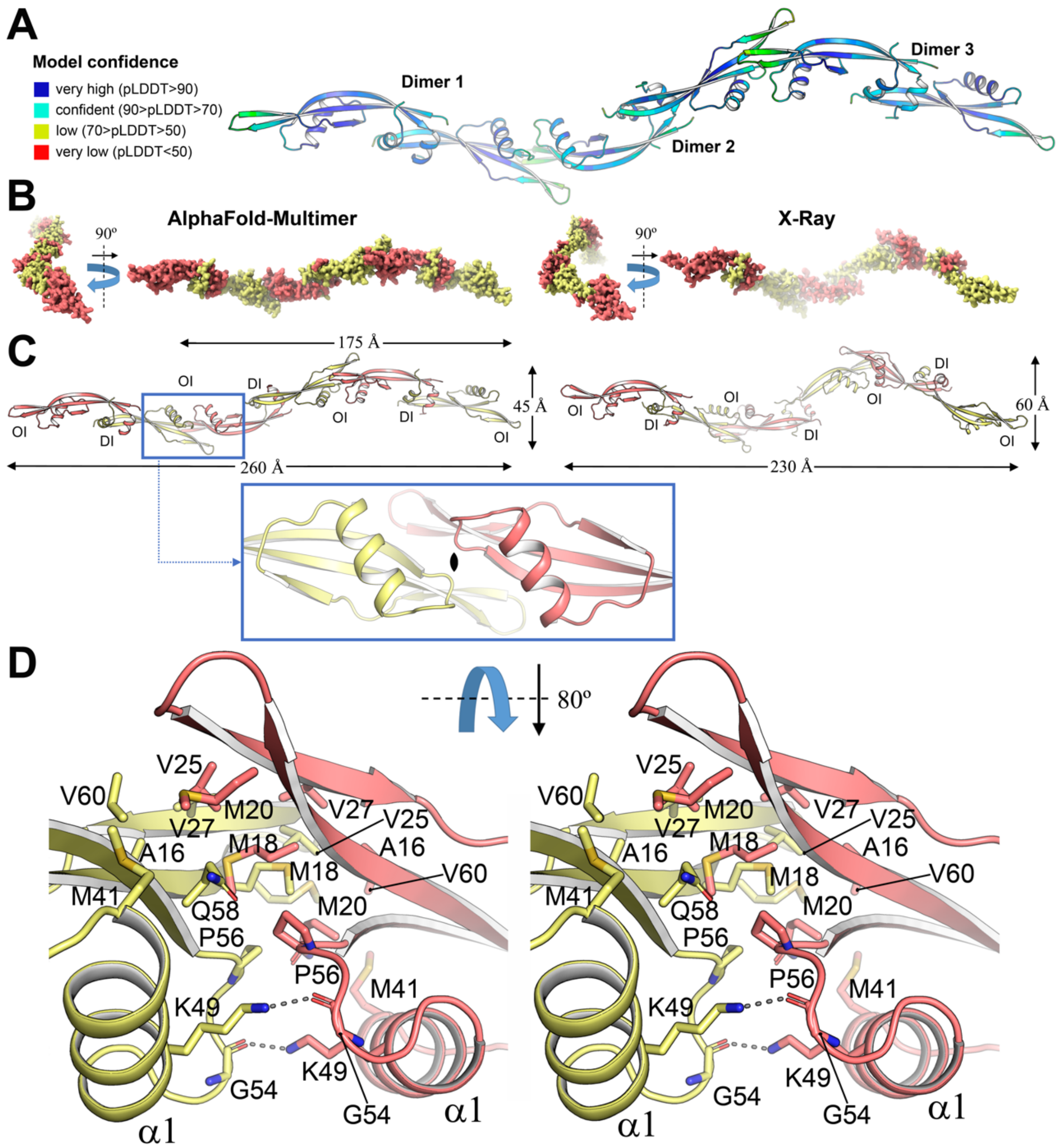

Supplementary Fig. 7. AF-Multimer structure prediction of the p6CΔ20 oligomer. (**A**) The predicted structure, depicted as a cartoon representation, reveals the arrangement of up to six protein monomers in the p6CΔ20 oligomer, with color coding indicating model confidence. (**B**) 90º views of the p6CΔ20 superhelix predicted by AF-Multimer (left panel) and its comparison with the p6CΔ20 oligomer structure obtained by X-ray crystallography (right panel). The surface of both oligomers is displayed. Each monomer within a dimer is color-coded, with salmon representing Monomer 1 and pale yellow representing Monomer 2. (**C**) Detailed depiction of the oligomeric arrangement of the p6CΔ20 filament predicted by AF-Multimer (left panel), along with a comparison to the X-ray crystallography-derived p6CΔ20 oligomer structure (right panel). Both cases are shown as lateral views of the protein filament in cartoon representation. The lower panel offers a zoomed-in view of the boxed area depicted in the upper left panel, highlighting the presence of a two-fold symmetry axis. DI, dimerization interface; OI, oligomerization interface. (**D**) Wall-eye stereo view of the predicted OI, focusing on the symmetrical interactions between Monomer 1 (salmon) and Monomer 2 (pale yellow). This view corresponds to the boxed area displayed in panel C after an 80º rotation. The capped sticks represent the relevant residues involved in oligomerization. Notably, the prediction suggests that the OI is primarily hydrophobic, but there is a single polar contact observed between K49 and G54, which is depicted as gray dotted lines.

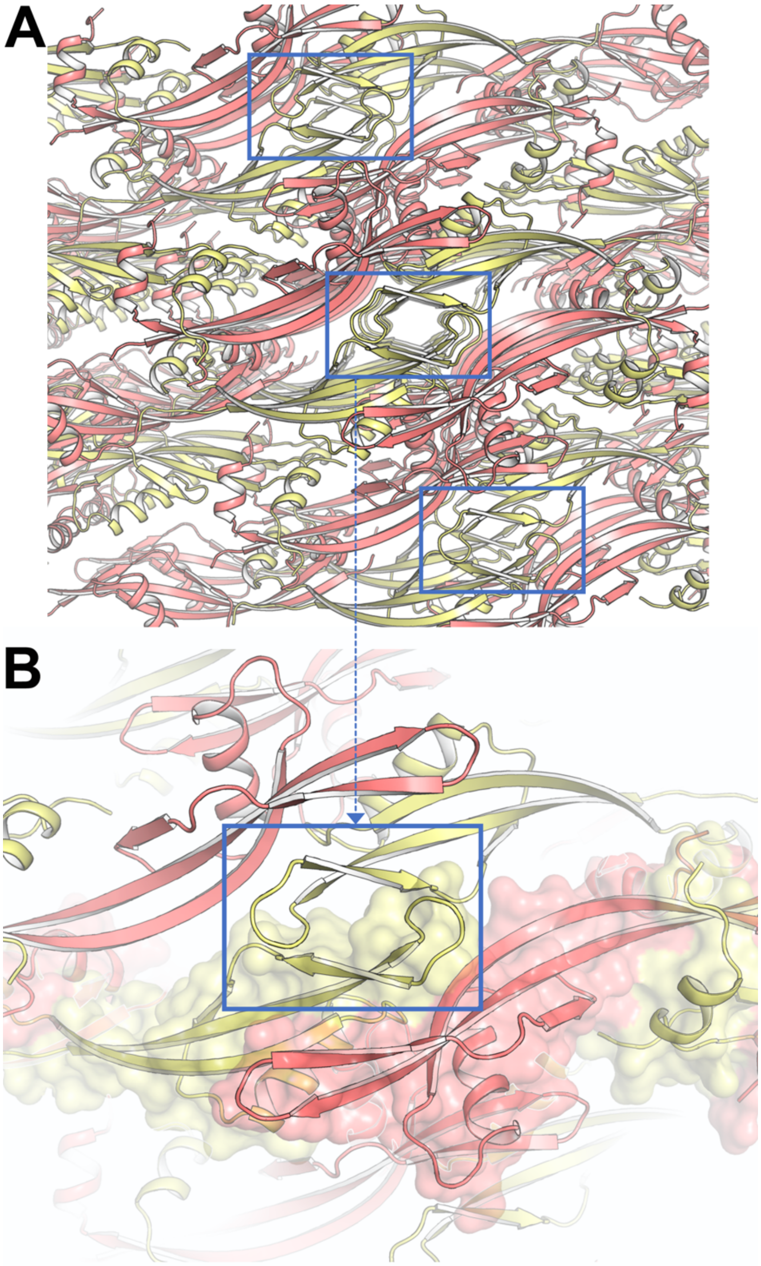

Supplementary Fig. 8. **Interactions involving the β-hairpins from neighboring fibers.** (**A**) Cartoon representation of the molecular packing observed in the p6CΔ20 crystals, in which each monomer within a dimer is color-coded, with salmon representing Monomer 1 and pale yellow representing Monomer 2. Notably, contacts between adjacent fibers primarily involve the β-hairpins, specifically from Monomer 2, as indicated by the boxed regions. (**B**) A zoomed-in view of the indicated boxed area depicted in the upper A panel (in which the surface of one of the fibers is visualized) emphasizing the interaction between two β-hairpins, each originating from separate protein fibers.

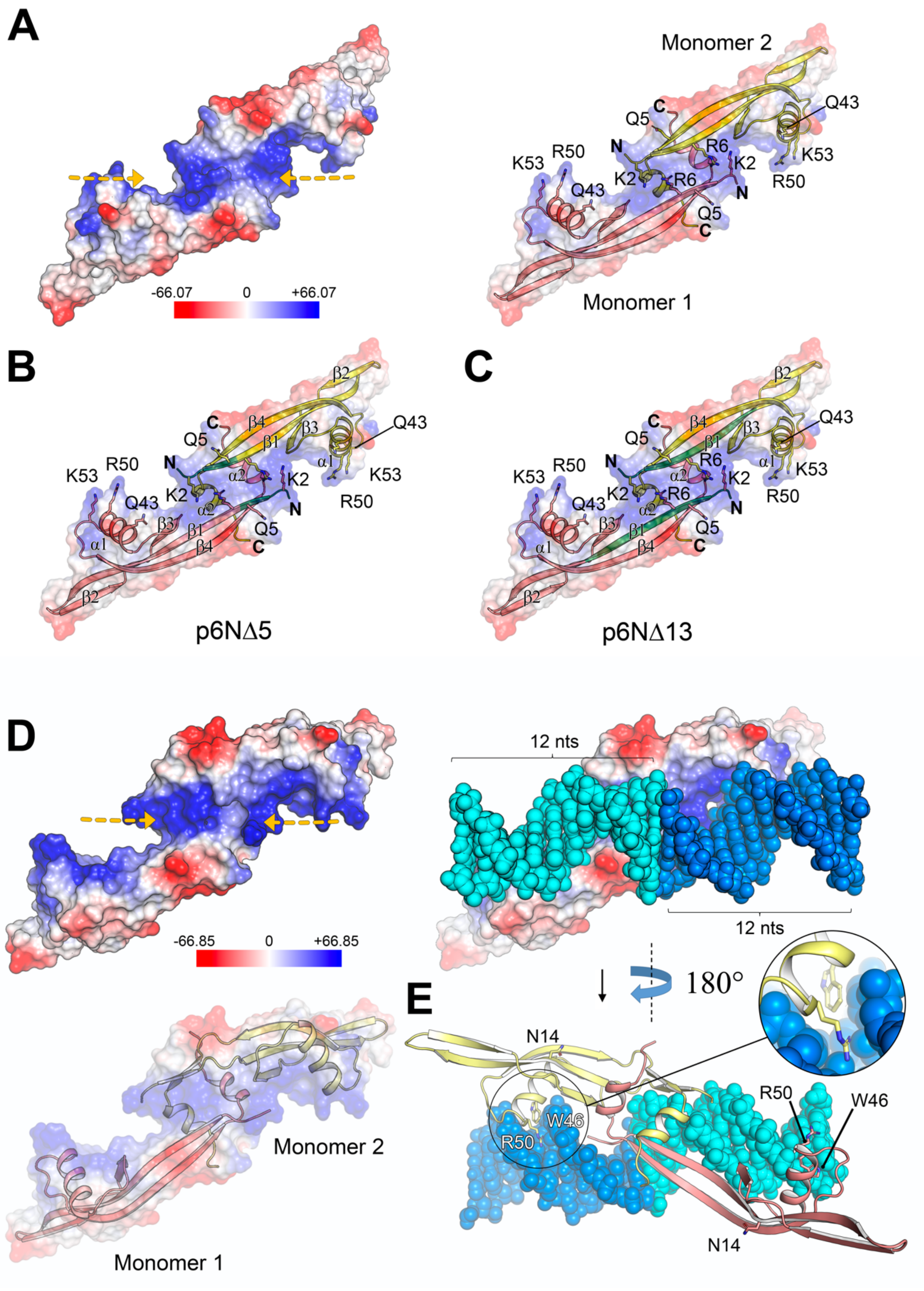

Supplementary Fig. 9. Localization of residues involved in the formation of the basic patch within the p6 dimer and mutations impacting DNA binding. (**A**) The left panel illustrates the Poisson-Boltzmann electrostatic-potential surface of the predicted p6CΔ20 dimer, with the color key representing positive charges in blue and negative charges in red. The range of the color bar is ± 66.07 kT/e. The right panel presents the same representation of the p6CΔ20 dimer, with each monomer (Monomer 1 in salmon and Monomer 2 in pale yellow) depicted in cartoon format. Residues contributing to the basic patch are shown as capped sticks and labeled. The yellow dashed line highlights the basic patch formed in the p6 dimer, which serves as the binding site for dsDNA. N, amino-terminus; C, carboxy-terminus. (**B**) Same panel as the one shown in A (right), highlighting the position of the deletion affecting the first 5 N-terminal

residues (mutant p6NΔ5), colored in green, on the structure of the predicted p6CΔ20 dimer. The secondary structure elements are labeled. (**C**) Similar to panel B, this panel highlights the location of the deletion affecting the first 13 N-terminal residues (p6NΔ13), colored in green. (**D**) The left panel illustrates the Poisson-Boltzmann electrostatic-potential surface of the p6CΔ20 dimer complexed with DNA after MD simulations, with the color key representing positive charges in blue and negative charges in red. The range of the color bar is ± 66.85 kT/e. The lower panel presents the same representation of the p6CΔ20 dimer shown in the upper D left panel with each monomer (Monomer 1 in salmon and Monomer 2 in pale yellow) depicted in cartoon format. Right panel shows the same protein dimer displayed in the left panel bound to a 24 bp dsDNA molecule, with the centers of the two monomer binding sites positioned 12 bp apart. The dsDNA is accommodated within the basic patch generated by the p6CΔ20 dimer. The DNA molecule is depicted as spheres, with the first half (spanning 12 nucleotides) colored in turquoise and the second half in dark blue. (**E**) This panel is a 180° rotated view (along the indicated axis) of panel D (right), excluding the surface representation. The p6CΔ20 dimer is depicted in cartoon format, with Monomer 1 in salmon and Monomer 2 in pale yellow. Residues Q14, W46 and R50 are shown as capped sticks (see main text for details).

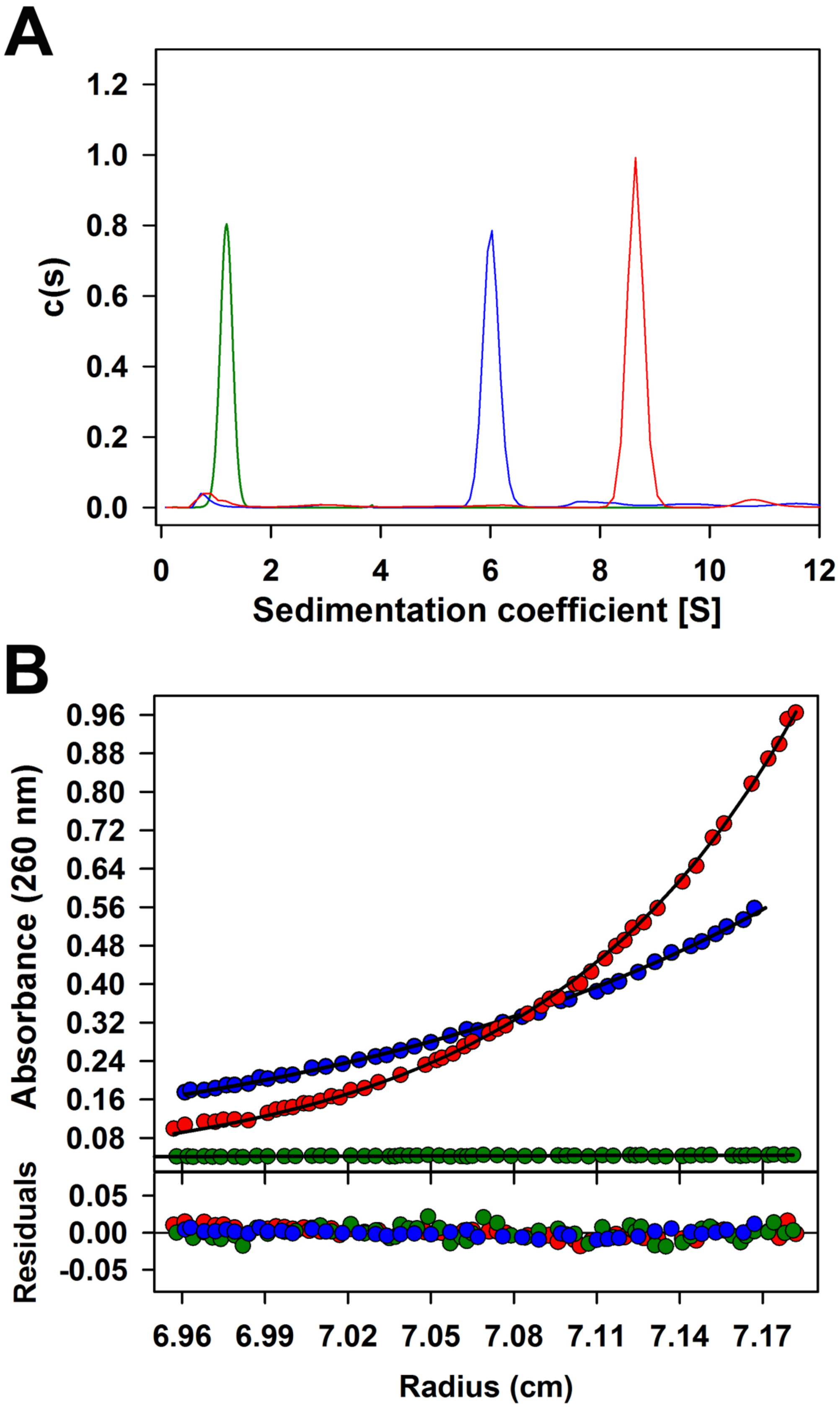

Supplementary Fig. 10. **Sedimentation velocity (SV) and sedimentation equilibrium (SE) experiments conducted to analyze the p6CΔ20–L complex.** Protein p6CΔ20 (5 µM, green), L (0.1 µM, blue), and p6CΔ20–L mixture (red). (**A**) Sedimentation coefficient distributions, *c*(*s*), obtained from SV assays at 42,000 rpm with Rayleigh interference, for p6CΔ20 alone, and absorbance at 260 nm for DNA-L alone or DNA-L in the presence of p6CΔ20, showing the shift in the s-value of p6CΔ20-DNA complex (red trace)

relative to the corresponding DNA–L alone (blue trace). (**B**) Concentration gradients obtained by SE at 5,000 rpm and 260 nm. Continuous lines represent the best-fit single-species model that accounts for the experimental data (circles) as described in the Materials and Methods section. The lower plot shows the difference between experimental data and estimated values for the single species model (residuals).

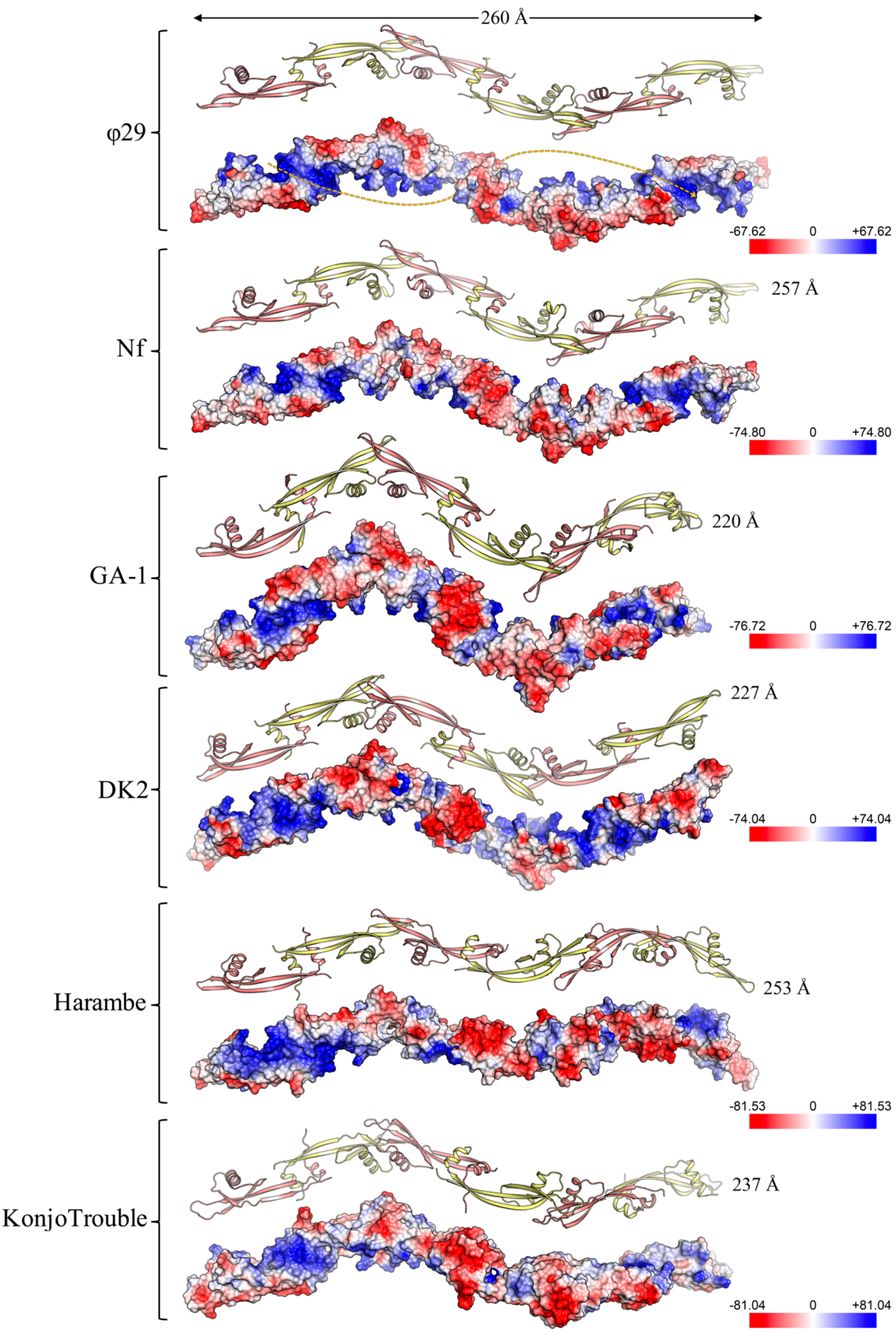

Supplementary Fig. 11. AF-Multimer predictions for the p6 filaments of the indicated φ29-relatives. For each phage, the upper panel shows a cartoon representation of the p6 superhelix comprising three protein dimers in which each monomer from a dimer is colored in salmon (monomer 1) and pale yellow (monomer 2). The lower panel presents the Poisson-Boltzmann electrostatic-potential surface maintaining the same orientation as the upper panel. The color key is blue, positive and red, negative. The fundamental

basic path that surrounds the p6 oligomer along its longitudinal axis, has been highlighted with a yellow dashed line for the particular case of phage φ29. The black arrow signifies the longitudinal extent traveled by three dimers of the protein superhelix. In all other cases, this distance is indicated to the right of each filament, underscoring the unique features of p6 filaments in diverse φ29-related phages.

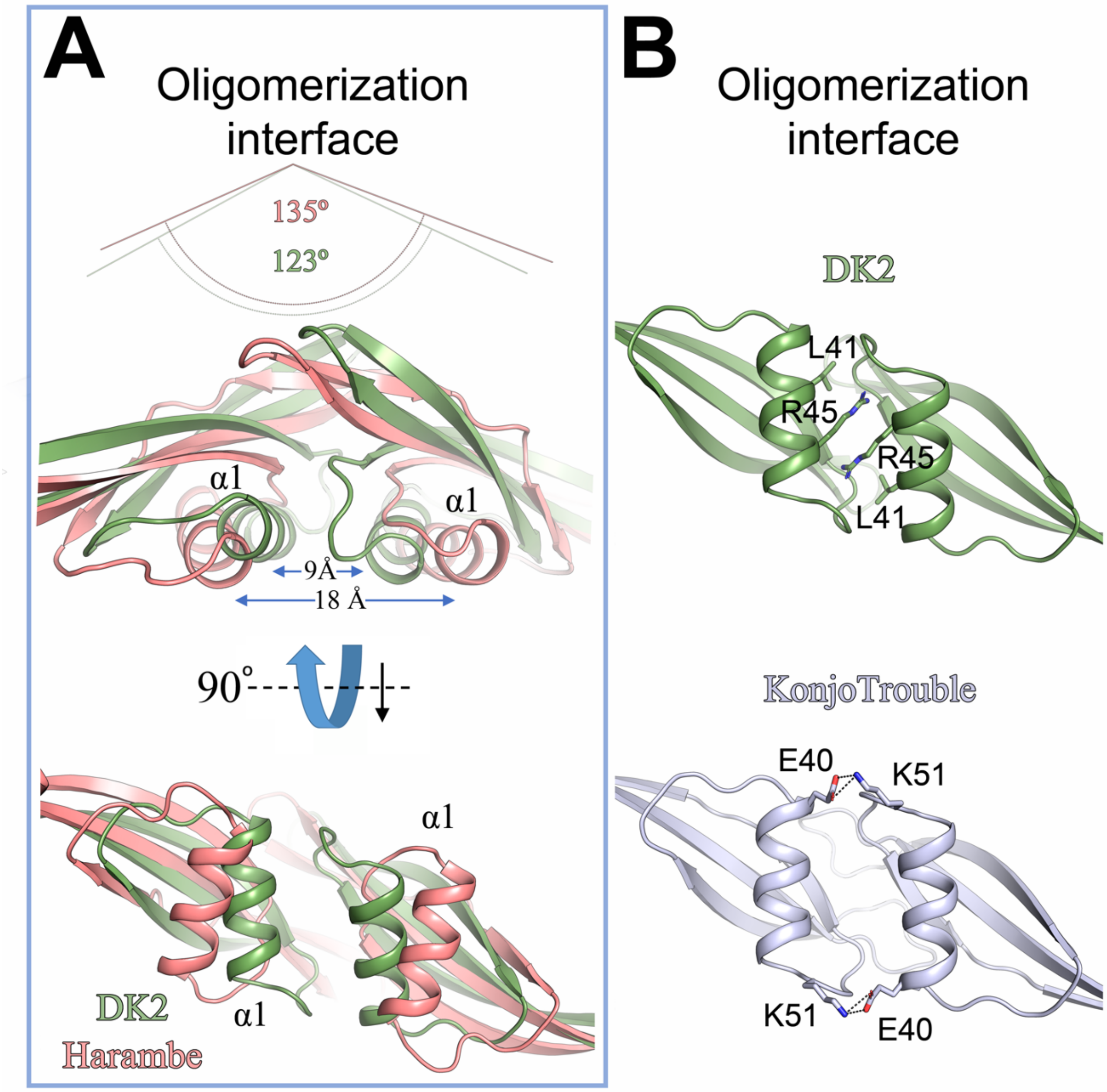

Supplementary Fig. 12. Structural variations are evident in the AF-predicted oligomerization interphase (OI) among different phages within the *Salasvirus* genus. (**A**) The predicted p6 filament OI of DK2 (depicted in green cartoon) and Harambe (depicted in salmon cartoon) phages are superposed to illustrate the structural differences. The two views are related by a 90º rotation around the indicated axis. The angle between the β-hairpins varies among different φ29 relatives, resulting in a shorter or longer distance between the α1 helices. (**B**) In the upper panel (depicted in green cartoon), the thigh packing between the α1 helices at the OI in the DK2 phage is shown, involving the side chains of L41 and R45 (represented by capped sticks). The lower panel (depicted in pale purple cartoon) displays the same view from phage KonjoTrouble, wherein intramolecular salt bridges between E40 and K51 are depicted by black dashed lines.

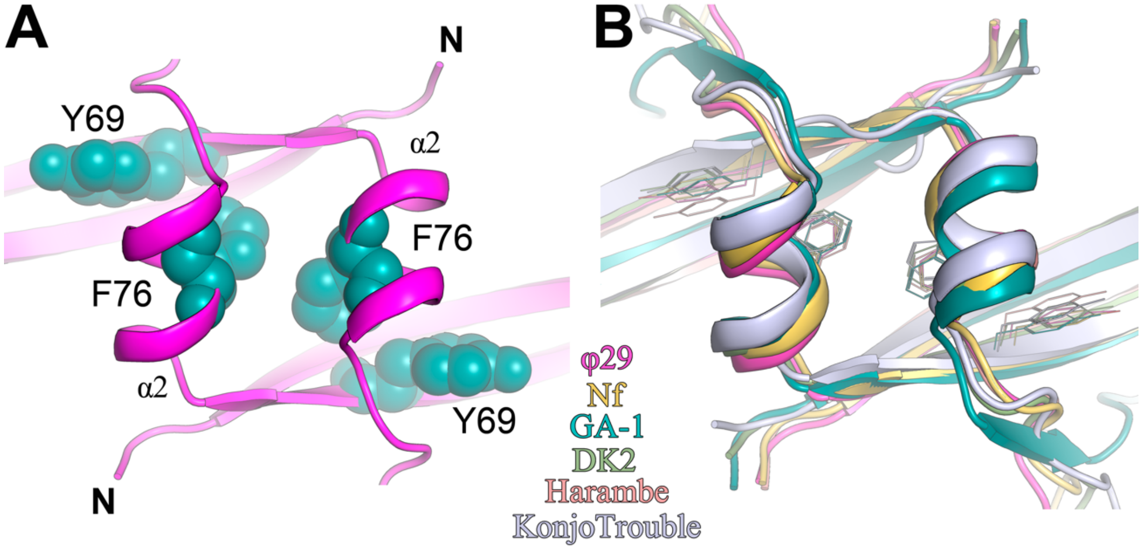

Supplementary Fig. 13. **Hydrophobic patch is observed at the dimerization interphase (DI).** (**A**) The DI of the φ29 protein p6 is highlighted (pink cartoon), specifically focusing on residues Y69 and F76 (depicted as green spheres). (**B**) The DI of the indicated phages is structurally superposed, and the corresponding residues shown in panel A are depicted using a lines representation (refer to the main text for further details).

MOVIE S1: Morphing generated with iMODS showing structural changes between the two monomers that are found in the crystallographic structure of the p6CΔ20 dimer.

MOVIE S2: The dynamic behavior of the p6 oligomer, as found in the X-ray crystal structure, was simulated in aqueous solution for 320 ns in the absence of any restraints by making use of an ensemble consisting of the p6 dimer from the asymmetric unit flanked on each longitudinal side by a symmetry-related dimer (cartoon representation, monomers colored as in the main text). The movie shows the concatenated snapshots (one for every 5 ns) from the 230-255 ns period of the unrestrained MD simulation (upon best-fit *rmsd* superposition of the middle dimer) and, at the very end, a still picture of all superimposed frames to show the inherent flexibility of the p6 fiber.

MOVIE S3: MEP surface on the crystallographic p6CΔ20 superhelix. The p6CΔ20 superhelix as observed in the crystal lattice is depicted in cartoon where each monomer from a dimer is colored in salmon (Monomer 1) and pale yellow (Monomer 2). Residues situated along the basic path are shown in stick representation. The MEP surface of the p6CΔ20 oligomer is also shown.

MOVIE S4: MEP surface on the p6CΔ20 superhelix as predicted by AF-Multimer. The p6CΔ20 superhelix as observed in the crystal lattice is depicted in cartoon where each monomer from a dimer is colored in salmon (Monomer 1) and pale yellow (Monomer 2). Residues situated along the basic path are shown in stick representation. The MEP surface of the predicted p6CΔ20 oligomer is also shown.

MOVIE S5: Representative time frame of the simulation of the p6CΔ20–DNA complex using unrestrained molecular dynamics for 350 ns. The DNA concatemer is displayed as a space-fill model colored in pale cyan whereas a ribbon representation was used for the p6 dimers, with each monomer colored as in the main text.

MOVIE S6: Representative snapshot depicting the p6CΔ20–DNA complex and showing the DNA concatemer as a space-fill model uniformly colored in pale cyan at the beginning. The DNA CPK-colored spheres thereafter correspond to the TCGA sites that become hypersensitive to DNase I upon binding of p6. DNase I is then shown as an enveloped cartoon colored in emerald bound at the strong hypersensitive sites, which are located opposite to the p6 binding site on the DNA major groove and display a wider minor groove that favors phosphodiester cleavage. Calcium and magnesium atoms are depicted as yellow and purple spheres, respectively.

References:

1. Poirot,O., Suhre,K., Abergel,C., O'Toole,E. and Notredame,C. (2004) 3DCoffee@igs: a web server for combining sequences and structures into a multiple sequence alignment. *Nucleic Acids Res*, **32**, W37-40.
2. Gouet,P., Courcelle,E., Stuart,D.I., Métoz,F. and Metoz,F. (1999) ESPript: analysis of multiple sequence alignments in PostScript. *Bioinformatics*, **15**, 305–308.
3. Kabsch,W. and Sander,C. (1983) Dictionary of protein secondary structure: pattern recognition of hydrogen-bonded and geometrical features. *Biopolymers*, **22**, 2577–2637.
4. Katoh,K. and Standley,D.M. (2013) MAFFT multiple sequence alignment software version 7: improvements in performance and usability. *Mol Biol Evol*, **30**, 772–780.
5. Darriba,D., Taboada,G.L., Doallo,R. and Posada,D. (2012) jModelTest 2: more models, new heuristics and parallel computing. *Nature Methods 2012 9:8*, **9**, 772–772.
6. Edler,D., Klein,J., Antonelli,A. and Silvestro,D. (2021) raxmlGUI 2.0: A graphical interface and toolkit for phylogenetic analyses using RAxML. *Methods Ecol Evol*, **12**, 373–377.
7. Letunic,I. and Bork,P. (2019) Interactive Tree Of Life (iTOL) v4: recent updates and new developments. *Nucleic Acids Res*, **47**.

8. Ashkenazy,H., Abadi,S., Martz,E., Chay,O., Mayrose,I., Pupko,T. and Ben-Tal,N. (2016) ConSurf 2016: an improved methodology to estimate and visualize evolutionary conservation in macromolecules. *Nucleic Acids Res*, **44**, W344–W350.
9. Abril,A.M., Salas,M. and Hermoso,J.M. (2000) Identification of residues within two regions involved in self-association of viral histone-like protein p6 from phage φ29. *Journal of Biological Chemistry*, **275**, 26404–26410.
10. Otero,M.J., Lázaro,J.M. and Salas,M. (1990) Deletions at the N terminus of bacteriophage φ29 protein p6: DNA binding and activity in φ29 DNA replication. *Gene*, **95**, 25–30.
11. Otero,M.J. and Salas,M. (1989) Regions at the carboxyl end of bacteriophage phi 29 protein p6 required for DNA binding and activity in phi 29 DNA replication. *Nucleic Acids Res*, **17**, 4567–4577.
